# An Eye-Tracking Study of Statistical Reasoning With Tree Diagrams and 2 × 2 Tables

**DOI:** 10.3389/fpsyg.2019.00632

**Published:** 2019-05-15

**Authors:** Georg Bruckmaier, Karin Binder, Stefan Krauss, Han-Min Kufner

**Affiliations:** ^1^Department of Secondary Education, University of Education, University of Applied Sciences and Arts Northwestern Switzerland, Windisch, Switzerland; ^2^Mathematics Education, Faculty of Mathematics, University of Regensburg, Regensburg, Germany

**Keywords:** Bayesian reasoning, eye tracking, 2 × 2 table, tree diagram, natural frequencies, probabilities

## Abstract

Changing the information format from probabilities into frequencies as well as employing appropriate visualizations such as tree diagrams or 2 × 2 tables are important tools that can facilitate people’s statistical reasoning. Previous studies have shown that despite their widespread use in statistical textbooks, both of those visualization types are only of restricted help when they are provided with probabilities, but that they can foster insight when presented with frequencies instead. In the present study, we attempt to replicate this effect and also examine, by the method of eye tracking, *why* probabilistic 2 × 2 tables and tree diagrams do not facilitate reasoning with regard to Bayesian inferences (i.e., determining what errors occur and whether they can be explained by scan paths), and *why* the same visualizations are of great help to an individual when they are combined with frequencies. All ten inferences of *N* = 24 participants were based solely on tree diagrams or 2 × 2 tables that presented either the famous “mammography context” or an “economics context” (without additional textual wording). We first asked participants for marginal, conjoint, and (non-inverted) conditional probabilities (or frequencies), followed by related Bayesian tasks. While solution rates were higher for natural frequency questions as compared to probability versions, eye-tracking analyses indeed yielded noticeable differences regarding eye movements between correct and incorrect solutions. For instance, heat maps (aggregated scan paths) of distinct results differed remarkably, thereby making correct and faulty strategies visible in the line of theoretical classifications. Moreover, the inherent structure of 2 × 2 tables seems to help participants avoid certain Bayesian mistakes (e.g., “Fisherian” error) while tree diagrams seem to help steer them away from others (e.g., “joint occurrence”). We will discuss resulting educational consequences at the end of the paper.

## Introduction

**FIGURE 1:**
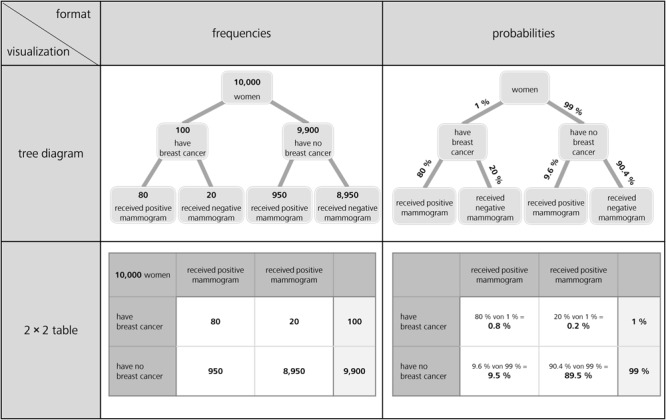
Tree diagrams (above) and 2 × 2 tables (below), both with frequencies (left) and with probabilities (right) for the mammography context (figure adapted from Binder et al., 2015).

**FIGURE 2:**
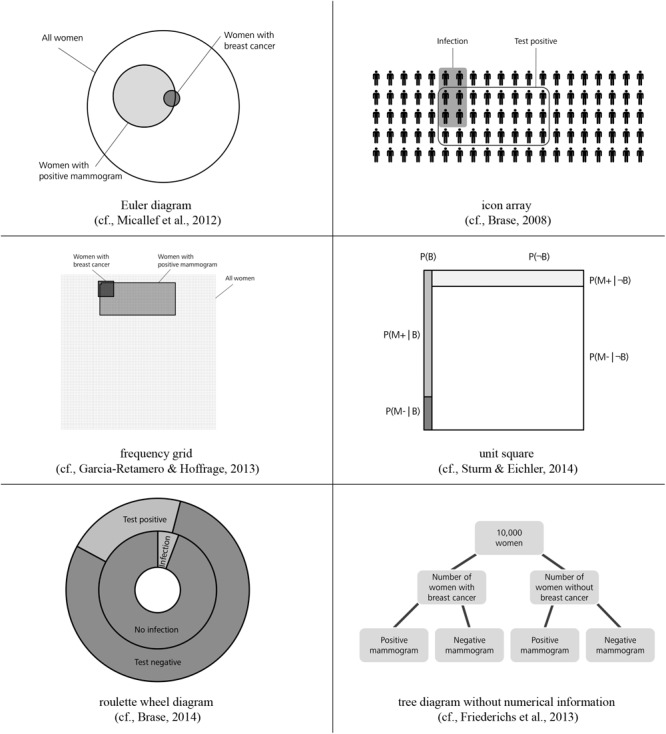
Alternative visualizations (figure from Binder et al., 2015).

It is relevant to one’s understanding of statistical situations involving two binary uncertain events (e.g., being ill: yes/no; medical test: positive/negative) whether the information is presented in probabilities (e.g., “80%”) or in natural frequencies (e.g., “8 out of 10”; Gigerenzer and Hoffrage, 1995). In the case of what is known as Bayesian reasoning situations, a meta-study found that the change of probabilities in natural frequencies substantially increases performance rates (McDowell and Jacobs, 2017; see also Barbey and Sloman, 2007). In Bayesian reasoning situations concerning medical contexts, the prevalence (*a priori* probability) of a disease is usually given, as well as the sensitivity and false-alarm rate of a medical test (see section Statistical Situations Based on Two Binary Events for a detailed theoretical distinction between Bayesian and non-Bayesian reasoning situations). Furthermore, a good deal of the literature demonstrates that visualizations can also foster insight into Bayesian reasoning or in statistical thinking in general (Yamagishi, 2003; Steckelberg et al., 2004; Binder et al., 2015; see also Figure 1, 2). In cognitive psychology—because of their relevance in real-world medical and legal decision-making (Hoffrage and Gigerenzer, 1998; Hoffrage et al., 2000; Fenton et al., 2016; Operskalski and Barbey, 2016)—Bayesian inferences stand firmly in the foreground of discussions about statistical reasoning.

In the field of statistics education, secondary school and university students have to assess and understand *all* probabilities concerning situations involving two binary events such as conjoint probabilities or (non-inverted) conditional probabilities (in such situations, 16 different probabilities can be considered, see section Statistical Situations Based on Two Binary Events). Thus in statistics classes taught at secondary schools or universities, a Bayesian inference is often treated as merely a (complicated) special case of conditional probability.

Regarding visualizations, in Germany but also in many other countries, tree diagrams and 2 × 2 tables are particularly widely implemented in textbooks on probability (see Figure 1; e.g., Eisentraut et al., 2008; Freytag et al., 2008; Schmid et al., 2008; Weber et al., 2018), most likely because both visualizations explicitly contain numbers and can be constructed easily by students based on typical problem wordings (neither of which is the case for, e.g., Euler diagrams or similar visualizations that rely on geometrical areas; see Figure 2; Weber et al., 2018). However, when the visualizations are equipped with probabilities (which in the classroom is most often the case), students unfortunately seem to struggle regardless of which of the two visualizations is used—especially concerning the notorious Bayesian inferences. Binder et al. (2015) could demonstrate that although German high school students are pretty much familiar with both visualizations, they cannot exploit tree diagrams or 2 × 2 tables with probabilities for respective inferences, and that the situation only changes when both visualizations are presented with frequencies (see Figure 1).

The study detailed in this paper attempts to replicate format effects concerning visualizations and goes one step further by investigating corresponding cognitive processes with the method of eye tracking. We expect with this method to be able to identify and describe typical (correct) solution strategies on the one hand, and on the other to explain specific errors frequently made by the participants. Thus our study investigates the intriguing question of why so many people struggle with probabilistic reasoning (including Bayesian), even when the widely prominent tree diagrams and 2 × 2 tables visualize the situation for them. What is wrong with these visualizations? And how do scan paths change when both visualizations are instead given with frequencies? Despite multiple calls for its use (Verschaffel et al., 2016; McDowell and Jacobs, 2017), the method of eye tracking has been applied only a few times thus far within the framework of statistical reasoning (Cohen and Staub, 2015; Reani et al., 2017; Lehner and Reiss, 2018), and not at all for analyzing format differences concerning both widely applied visualizations.

It has to be noted that most research in the field of cognitive psychology or statistics education—with a strong focus on the special case of Bayesian inferences, especially in cognitive psychology—is concerned with attempts to boost performance, for instance by changing the information format or presenting additional visualizations (see, e.g., the recent meta-analysis by McDowell and Jacobs, 2017), by implementing trainings (e.g., Sedlmeier and Gigerenzer, 2001; Steckelberg et al., 2004), or by theoretically explaining the benefit of certain tools (e.g., the discussion between proponents of the ecological rationality approach and the nested sets approach, Hoffrage et al., 2000; Pighin et al., 2016). With mathematics education in mind, the present research is in line with recent studies also conducted by our research group that look at the other side of the coin of statistical reasoning: when and why teaching fails. For instance, by focusing on participants who failed in Bayesian inferences *although* the information was displayed in terms of the favored frequencies, Weber et al. (2018) could demonstrate that due to a “fixed mindset,” many of these students translated the given natural frequencies “back” into probabilities, with the consequence that they were not able to solve the task.

In the first theoretical section of the paper, we will show that Bayesian inferences are only a special case in situations with two binary uncertain events, and examine which other probabilities are regularly covered in teaching at secondary school and university. We will then explain why tree diagrams and 2 × 2 tables are both widely implemented worldwide in the actual teaching of statistics, and what is already known about typical errors that are made with regard to inferences based on those two visualizations. In this way, the rationale of our present approach combines the concept of natural frequencies and the focus on *Bayesian* reasoning from cognitive psychology with a consideration of all 16 probabilities and the choice to utilize tree diagrams and 2 × 2 tables in typical statistics education materials used at secondary school and university.

## Statistical Thinking

### Statistical Situations Based on Two Binary Events

Bayesian situations usually refer to two binary uncertain events such as a state of health (being ill vs. not being ill) and a medical test result (e.g., positive vs. negative). In secondary school, and especially with younger children, the respective events might, for instance, be the gender of a child (female vs. male) and a certain personality trait (e.g., loves sports vs. does not love sports). In general, in such situations, 16 different probabilities can be theoretically considered, which we will illustrate with the case of the famous mammography context (that will also be applied later on as one of the two contexts in our empirical study). The mammography context contains two events, each with binary values (B: having breast cancer; B: not having breast cancer; M+: positive mammogram; M-: negative mammogram), which allows for the consideration of the following probabilities:

Four probabilities taking just one event into account (marginal probabilities):

P(B), P(¬B), P(M+), P(M-),

with P(¬B) = 1 - P(B) and P(M-) = 1 - P(M+)

Four conjoint probabilities:

P(B ∩ M+), P(¬B ∩ M+), P(B ∩ M-), P(¬B ∩ M-)

Eight conditional probabilities:

P(M+|B), P(M+|¬B), P(M-|B), P(M-|¬B), P(B|M+), P(B|M-), P(¬B|M+), P(¬ B|M-)

Note that thus far, no task is given, and it is possible to describe these situations in general without the need to decide on a special inference (consequently, in the following we will strictly distinguish between the “mammography situation” *per se* and the corresponding problem/task posed). Respective inferences often require—in cognitive psychology and in the teaching of statistics as well—deducing a certain probability when at least three other probabilities are given. The most prominent examples are Bayesian inferences that involve the inversion of a given conditional probability. For instance:

Mammography problem (probability format):

The probability of breast cancer (B) is 1% for a woman of a particular age group who participates in a routine screening (P(B)). If a woman who participates in a routine screening has breast cancer, the probability P(M+|B) is 80% that she will have a positive mammogram (M+). If a woman who participates in a routine screening does not have breast cancer (B), the probability P(M+|*B)* is 9.6% that she will have a false-positive mammogram.

What is the probability that a woman who participates in a routine screening and has a positive mammogram has breast cancer?

The required Bayesian inference is an “inversion” in the sense that a conditional probability P(M+|B) is given and the “inverse” conditional probability P(B|M+) has to be assessed in order to “update” an *a priori* estimation [in this case P(B)]. In the light of this new evidence, Bayes’ theorem yields:

(1) $$\begin{array}{c} P(B|M+)=\frac{P(M+|B)P\left(B\right)}{P(M+|B)P\left(B\right)+P(M+|\neg B)P\left(\neg B\right)}\\ =\frac{80\%\cdot 1\%}{80\%\cdot 1\%+9.6\%\cdot 99\%}=7.8\% \end{array}$$

It is well known that such solutions may be counterintuitive (especially when extreme base rates like 1% are given) and that most people (even experts like physicians) have difficulty estimating such probabilities. In the meta-analysis by McDowell and Jacobs (2017), only 4% of the participants were able to come up with correct answers concerning such inferences. However, in addition to these problematic Bayesian inversions, the assessment of conjoint probabilities (e.g., Fiedler, 2000) can also be difficult.

### Information Formats: Probabilities vs. Frequencies

Nevertheless, situations like these can actually be taught to very young children who are not even aware of the concept of conditional probability (or probabilities in general). In German secondary schools, for instance, such situations are introduced to children as young as 10, with absolute numbers concerning a set of persons (or objects) provided, each of them having (or not having) two certain characteristics. For instance, there may be 100 students, and the two characteristics might be gender (male or female) and wearing glasses (or not). Note that when a certain sample is given, all of the 16 probabilities mentioned above can be expressed in absolute numbers that describe specific subsets. The fact that absolute numbers are much easier to grasp is exploited by the concept of *natural frequencies* (Gigerenzer and Hoffrage, 1995), which even foster insight into Bayesian inferences. Natural frequencies combine two absolute frequencies, as illustrated in the mammography problem:

Mammography problem (natural frequency format):

100 out of 10,000 women of a particular age group who participate in a routine screening have breast cancer. 80 out of 100 women who participate in a routine screening and have breast cancer will have a positive mammogram. 950 out of 9,900 women who participate in a routine screening and have no breast cancer will have a false-positive mammogram.

How many of the women who participate in a routine screening and receive positive mammograms have breast cancer?

Substantially more people are now able to find the correct solution to the problem (which is “80 out of 1,030”) because the solution becomes more obvious and the calculation is easier. In the meta-analysis by McDowell and Jacobs (2017), frequency versions of Bayesian reasoning problems can be solved on average by 24% of participants across studies and contexts. Even in more complex Bayesian problems, such as in situations involving more than one medical test or unclear test results, frequencies help people in their decision-making processes (Hoffrage et al., 2015b; Binder et al., 2018). In the last 20 years, an abundance of studies has shown the facilitating effect of frequencies for many different kinds of populations: physicians, patients, judges in court, managers, university and high school students, and even young children (Gigerenzer and Hoffrage, 1995; Hoffrage et al., 2000; Zhu and Gigerenzer, 2006; Siegrist and Keller, 2011; Hoffrage et al., 2015a; McDowell and Jacobs, 2017). Weber et al. (2018), on the other side, shed light on the question of why (on average) 76% of participants still fail even though frequencies (instead of probabilities) are provided, finding that many participants translated the given frequencies back into (more complicated) probabilities.

Natural frequencies can be obtained both by natural sampling (Kleiter, 1994) or, alternatively, by actively translating given probabilities (e.g., “80%”) into expressions consisting of two absolute frequencies (e.g., “80 out of 100”). In our research—in contrast to some other scholars’ work (e.g., Spiegelhalter and Gage, 2015)—we consider natural frequencies as the superordinate concept for both *empirically sampled* and *expected* frequencies. While the latter constitute frequencies that are expected in the long run (cf. Hertwig et al., 2004; Spiegelhalter and Gage, 2015; case 2 in Woike et al., 2017), empirically sampled frequencies are derived from a natural sampling process (cf. Kleiter, 1994; Fiedler et al., 2000; cases 1 and 3 in Woike et al., 2017). Whereas empirically sampled frequencies can obviously deviate from the expected ones (but are still natural frequencies), expected frequencies fit perfectly into the teaching context (here, natural frequencies usually stem from imagining a specific sample).

Furthermore, it is not only natural frequencies of Bayesian tasks that can be considered natural frequencies. Of course, on the one hand it is possible to sample all of the 16 probabilities mentioned above in terms of natural frequencies (by natural sampling). And, on the other hand, if probabilities are given, all of them can actively be translated into natural frequencies as a didactical tool (by researchers, teachers, or clever students, who realize that only an arbitrary sample functioning as reference set has to be imagined first).

### Number-Based Visualizations: 2 × 2 Tables and Tree Diagrams

In their research articles, scholars often use 2 × 2 tables (Goodie and Fantino, 1996; Dougherty et al., 1999; Fiedler et al., 2000) or tree diagrams (Kleiter, 1994; Gigerenzer and Hoffrage, 1995; Mandel, 2014; Navarrete et al., 2014) to illustrate Bayesian reasoning situations to their peers. Both visualizations are also very prominent in the context of statistical education at secondary school and university. Interestingly, the effects of these visualizations on participants’ performance have only rarely been tested empirically thus far (for a discussion, e.g., see Binder et al., 2015). With the numbers from the mammography context above, there are generally four possible different visualizations of this kind (see Figure 1). The cause for the calculations in the cell at the below right is explained in issue 1 (see later in section Number-Based Visualizations: 2 × 2 Tables and Tree Diagrams).

Why are these visualizations so prominent, especially in the context of teaching? Note that in contrast to most other visual aids (see Figure 2), 2 × 2 tables and tree diagrams usually explicitly contain numerical information and, furthermore, both can be equipped with frequencies or with probabilities (Figure 1). The decisive advantage for teaching and learning, however, is that teachers and students can easily construct all of these visualizations themselves. Note that “non-numerical” visualizations such as Euler diagrams (e.g., Sloman et al., 2003; Brase, 2008; Micallef et al., 2012; Sirota et al., 2014b), roulette wheel diagrams (e.g., Yamagishi, 2003; Brase, 2014), or unit squares (Böcherer-Linder and Eichler, 2017), all of which are based on geometrical areas (Figure 2), require a substantial effort to be produced (i.e., sometimes the size of the specific areas needed for the visualizations can only be calculated when the task is already solved). Furthermore, it is not always convenient to display extreme base rates by a geometrical area. For instance, in a true-to-scale unit square, the prevalence of 1% would no longer be visible. Along the same lines, for displaying the mammography problem with an icon array (Brase, 2008, 2014; Sirota et al., 2014b; Zikmund-Fisher et al., 2014), which is based on small symbols instead of geometrical areas, the student (or teacher) would have to draw 10,000 icons.

It is important to note that, in principle, all visualizations appearing in Figure 1, 2 allow for the assessment of *all* of the 16 probabilities above (which is also true for all typical, purely textual formulations of Bayesian tasks). Furthermore, one can present not only “normal” tree diagrams or 2 × 2 tables, but also ones with highlighted branches or nodes (see Binder et al., 2018) or cells. Cognitive load theory (Sweller, 2003) would suggest that according to the signaling principle, highlighting the relevant branches, nodes, or cells might improve performance of participants (Mautone and Mayer, 2001; Mayer, 2008). Furthermore, a combination of textual and visual information could shed more light on the redundancy principle of multiple information sources, which is addressed in the cognitive load theory and the cognitive theory of multimedia learning (Mayer, 2005). The redundancy principle says, in short, that the elimination of any redundant information may enhance learning (see Sweller, 2003; Mayer, 2005) because of a reduction of the extraneous cognitive load (also see Discussion).

Concerning the four visualizations of Figure 1 that are widely used in teaching and that we will also implement in our empirical study (for the final stimuli, see Figure 4), some theoretical details have to be clarified:

(1) *2 × 2 tables cannot present conditional probabilities (only tree diagrams can)*:
  Concerning the probability format, it is obvious that the probabilities provided in a Bayesian task *cannot* be placed directly into a 2 × 2 table, since 2 × 2 tables contain *conjoint probabilities* but not conditional ones. Therefore, while the conditional probabilities given in a Bayesian task can be placed directly on the branches of a tree diagram, 2 × 2 tables principally display different pieces of information (see Figure 1).
  This feature of 2 × 2 tables makes them simpler (compared to tree diagrams) in terms of the calculations to be performed, at least for Bayesian inferences based on probabilities, because a part of the calculation has already been performed in order to complete the 2 × 2 table (as indicated in small letters in Figure 1 in the cell below right). Note that only a tree diagram with probabilities requires Bayesian calculations according to formula (1), while in 2 × 2 tables the following calculation is sufficient for the resulting conditional probabilities:

(2a) $$\begin{array}{c} P(B|M+)=\frac{P\left(B\cap M+\right)}{P\left(M+\right)}=\frac{0.8\%}{0.8\%+9.5\%}\approx 7.8\% \end{array}$$

Consequently, since Bayesian inferences imply the aspect of inversion, it is interesting to consider whether inferences based on 2 × 2 tables containing probabilities can be called “Bayesian” at all (e.g., Binder et al., 2015, but see the short menu in Gigerenzer and Hoffrage, 1995). Therefore, in our experiments only one marginal distribution is shown (see Figure 4) because displaying the other one in addition would allow simply to dividing the numbers in two cells for all conditional probabilities. Thus, inverted and non-inverted conditional probabilities could not be distinguished any longer.
(2) *Concerning 2 × 2 tables, scan paths (gaze behavior) should not depend on information format*:
  Concerning possible scan paths, it is important to note that, regarding 2 × 2 tables (see below in Figure 1), exactly the same cells would have to be inspected in both formats for all 16 possible inferences. In contrast, probabilities in tree diagrams are depicted at the branches and absolute frequencies in the nodes, thus requiring slightly deviating scan paths in the two formats. For the 2 × 2 table presented with frequencies of the mammography context, similar to formula (2a), two frequencies (instead of probabilities) have to be added to obtain the denominator in formula (2b):

(2b) $$\begin{array}{c} P(B|M+)=\frac{\#\left(B\cap M+\right)}{\#\left(M+\right)}=\frac{80}{80+950}\approx 7.8\% \end{array}$$

(3) *Frequentistic visualizations are more flexible than textual natural frequency versions*:
  Notably, both frequentistic visualizations (see left side in Figure 1) contain absolute frequencies, implying that natural frequencies of the type “x out of y” (i.e., natural frequencies always consist of two absolute frequencies) would have to be combined by first relating two absolute numbers (x and y) in any case. However, this necessity makes frequency visualizations flexible, since the absolute frequencies displayed in Figure 1 can be combined to multiple kinds of natural frequencies (e.g., “80 out of 100,” “100 out of 10,000,” “80 out of 10,000”).
(4) *2 × 2 tables and tree diagrams display more statistical information than textual wording*:
  Furthermore, it is striking that in all four visualizations (Figure 1), *more* numerical information is displayed than in the corresponding mammography wordings (specifically, statistical information on the respective counter events is included). However, concerning Bayesian inferences, this additional information can usually be disregarded.
(5) *Non-inverted vs. inverted (Bayesian) conditional probabilities*:
  Most importantly, with respect to Bayesian reasoning, tree diagrams (above in Figure 1) entail a specific order of subsetting: First, the sample is divided according to state of health, then according to test result (an inverse tree diagram can easily be imagined by first dividing the sample according to M+ and M-, and subsequently according to the state of health). In order to mirror this structure in the corresponding 2 × 2 tables, we deliberately presented only one of the two marginal distributions (in both formats, see Figure 4). As a consequence, we can distinguish in all four visualizations between “normal” conditional probabilities and inverse conditional probabilities in the following way: Non-inverted conditional probabilities (and frequencies as well) require a simple division of two pieces of information displayed (in the “probability tree,” the non-inverted conditional probabilities can even be taken directly from the lower branches). In contrast, as explicated above, the inversion of conditional probabilities (and thus Bayesian reasoning) requires more complex cognitive operations. Note that formulas (1) and (2a), based on the probability tree or the “probability 2 × 2 table,” and formula (2b), based on both frequentistic visualizations, *all* entail more operations than the simple division of two pieces of information.
(6) *2 × 2 tables and tree diagrams in secondary schools*:
  Finally, it has to be noted that the 2 × 2 table (with conjoint probabilities), the 2 × 2 table (with frequencies), and the tree diagram (with probabilities) are part of the German secondary school curriculum, whereas the “frequency tree” is not. However, (Bayesian) inferences based on both frequency visualizations seem to be much easier than those based on both probability visualizations (Binder et al., 2015), which brings into question the omnipresent application of the latter in the teaching of statistics. This emphasizes the schools’ challenge in teaching the intelligent reading of visualizations (i.e., the facets “read the data,” “read between the data,*”* and “read beyond the data” from Curcio, 1989).

### Error Strategies Detectable in Tree Diagrams and 2 × 2 Tables

Many statistics educators, but also the psychologists McDowell and Jacobs (2017) in their meta-analysis on Bayesian reasoning, stress the importance of investigating erroneous cognitive algorithms. This, of course, is true for teaching and learning mathematics in general (e.g., Krauss et al., 2008). But only a few studies have explicitly reported typical incorrect reasoning strategies concerning Bayesian inferences (for some exceptions, see Gigerenzer and Hoffrage, 1995; Steckelberg et al., 2004; Zhu and Gigerenzer, 2006; Eichler and Böcherer-Linder, 2018; Weber et al., 2018).

**FIGURE 3:**
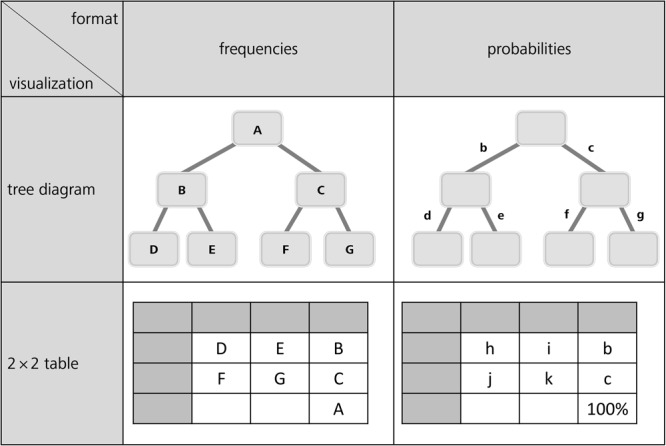
General tree diagrams (above) and 2 × 2 tables (below) provided with frequencies (left) or probabilities (right).

In order to gain insight into the cognitive problems that people encounter concerning Bayesian inferences and statistical thinking in general, a better understanding of typical errors is required. The few existing classifications of incorrect Bayesian strategies are summarized in Table 1. While Gigerenzer and Hoffrage (1995) describe the typical erroneous strategies based on probabilities, Zhu and Gigerenzer (2006) and Eichler and Böcherer-Linder (2018) choose an explanatory approach based on frequencies. To relate all types of errors to our four visualizations (Figure 1), we first display both kinds of classifications next to each other (Table 1). In doing so, we present the errors based on the notation shown in Figure 3 (uppercase letters stand for absolute frequencies while lowercase letters represent probabilities). Keep in mind that these letters will later on be used to denote respective areas of interest (AOIs).

**Table 1:** Correct solution and typical incorrect (Bayesian) strategies according to the correct solution “*D* out of *D* + *F*” in a typical Bayesian reasoning task (according to Gigerenzer and Hoffrage, 1995; Steckelberg et al., 2004; Zhu and Gigerenzer, 2006; Eichler and Böcherer-Linder, 2018).

	Frequencies	Probabilities
	(with *A, B, C, D, E, F, G*^∗^)	(with *b, c, d, e, f, g, h, i, j, k*^∗^)
**Correct solution (Bayesian)**	***D* out of (*D* + *F*)**	**(*b* ⋅*d*)/ (*b* ⋅*d* + *c* ⋅*f*)**

**Incorrect algorithm (non-Bayesian)**		
Joint occurrence (Gigerenzer and Hoffrage, 1995)	*D* out of *A*	*b* ⋅*d*
Fisherian (Gigerenzer and Hoffrage, 1995)/Representative thinking	*D* out of *B*	*d*
Likelihood subtraction (Gigerenzer and Hoffrage, 1995)	*(D* out of *B)* –(*F* out of *C)*	*d - f*
Base rate only (Gigerenzer and Hoffrage, 1995)/conservatism (Zhu and Gigerenzer, 2006)	*B* out of *A*	*b*
Evidence-only (Zhu and Gigerenzer, 2006)	(*D* + *F*) out of *A*	*b* ⋅*d* + *c* ⋅*f*
Pre-Bayes (Steckelberg et al., 2004; Zhu and Gigerenzer, 2006)	*B* out of (*D* + *F*)	Not applicable
Correct positive rate/false positive rate (Steckelberg et al., 2004)	(*D*/*B*) out of (*F*/*C*)	*d*/*f*

*^∗^*A, B, C*, etc., and *b, c, d*, etc. represent the pieces of statistical information in the respective visualization (see also Figure 3).*

Note, however, that the errors reported refer to the typical textual formulations of Bayesian reasoning tasks implemented (see, e.g., the wordings of the mammography problem in the probability and frequency formats in sections Statistical Situations Based on Two Binary Events and Information Formats: Probabilities vs. Frequencies). Gigerenzer and Hoffrage (1995) found the *joint occurrence* to be the most frequent erroneous strategy in Bayesian reasoning. Joint occurrence involves multiplying the *base rate b* and the *sensitivity d* (in frequencies: divide *D* by *A*) without considering the healthy people with positive test results (i.e., *c* and *f*; or correctly dividing *D* by *D+F*). According to the same authors, another frequently applied erroneous strategy is the *Fisherian* (or *representative thinking*, according to Zhu and Gigerenzer, 2006) strategy, in which one only takes the sensitivity *d* of the test as the answer (or in terms of frequencies: to calculate *D/B*). This error is widespread because it is tempting to confuse P(B|M+) with P(M+|B). Furthermore some participants used another wrong algorithm, which is called *likelihood subtraction* (Gigerenzer and Hoffrage, 1995), meaning erroneously to compute P(M+|B) – P(M+|–B). However, this wrong algorithm predominately occurs in probability versions and is rather unusual for natural frequency versions. A few other participants in that study (Gigerenzer and Hoffrage, 1995) only provided the *base rate b* as the solution of the Bayesian reasoning task, which in frequencies means dividing *B by A* (this error is called *conservatism* by Zhu and Gigerenzer, 2006). The authors also identified the error *evidence-only*, which is the proportion of people with positive test results [i.e., *c* and *f*; or, (*D*+*F*) out of *A*, respectively]. Furthermore, Zhu and Gigerenzer (2006) as well as Steckelberg et al. (2004) reported an error that is documented for frequency versions only, namely *pre-Bayes* (which means to incorrectly divide *B* by *D+F*). Finally, some participants also applied the erroneous strategy *correct positive rate/false positive rate* (Steckelberg et al., 2004).

Because visualizations could prevent specific misunderstandings or even block faulty algorithms, it is crucial to reconsider cognitive algorithms with respect to specific visualizations. For instance, the (Fisherian) confusion of P(A|B) with P(B|A) might occur less frequently with a tree diagram (compared to a text-only version) since tree diagrams emphasize the sequential character of the situation more. But even though different visualizations might help for very different reasons, they could also cause new errors that are not listed in Table 1. Certain new types of errors might occur according to cognitive load theory (Sweller, 2003) precisely because more information is presented in a tree diagram or in a 2 × 2 table than in a textual version of a Bayesian task. For instance, *E* and *G* or the corresponding probabilities *e* and *g* (cf. Figure 3) only appear in visualizations but not in typical wordings, and it is possible for people to erroneously make use of this statistical information in their calculations. It has to be noted that Steckelberg et al. (2004) mention incorrect Bayesian strategies associated with visualizations (tree diagrams and 2 × 2 tables), but do not discuss them in detail. Likewise, possible explanations of the beneficial effect of particular visualizations often remain theoretical (see, e.g., Khan et al., 2015).

For teaching statistics, just as for teaching mathematics in general, it is essential to be an expert on typical errors and on learners’ preconceptions (Shulman, 1986, 1987; Krauss et al., 2017). To this end, McDowell et al. (2018) call for a broader methodological approach that can identify typical incorrect Bayesian strategies. Johnson and Tubau (2015) and McDowell and Jacobs (2017) even explicitly suggest eye-tracking analyses of Bayesian reasoning strategies. As educators for future mathematics teachers, we are in addition interested in the pros and cons of visualizations regarding all 16 possible inferences, especially concerning the most frequently applied visualizations in the (German) context of teaching statistics in secondary schools and universities, namely 2 × 2 tables and tree diagrams.

In the second theoretical section of this paper, we will now focus on the method of eye tracking and how it has been used thus far concerning strategy detection in general but also with respect to statistical reasoning in particular. For this purpose, we introduce the design and results of three studies that are closest to the approach followed in the present article.

## Eye Tracking as a Method for Assessing Statistical Reasoning Strategies

### Research Techniques for Identifying Cognitive Processes

Most empirical studies on Bayesian reasoning (or statistical thinking in general) primarily focus on participants’ performance rates. However, neither performance rate nor reaction time can fully explain underlying reasoning processes. Verbal reports (or qualitative interviews) might be a path toward an identification of strategies (Robinson, 2001; Smith-Chant and LeFevre, 2003), but participants may have insufficient explicit knowledge to be able to theoretically reflect solution strategies (especially *post hoc*). Therefore, the think-aloud and write-aloud methods (van Someren et al., 1994; for write-aloud protocols on Bayesian reasoning, see Gigerenzer and Hoffrage, 1995) represent an alternative, requiring participants to report on their reasoning strategies simultaneously to their problem solving. However, although this method certainly offers valuable insight into the cognitive strategies that are employed in task processing, it obviously also affects the problem-solving itself.

In contrast, the method of *eye tracking*—a non-invasive measurement of eye movements relative to the head and the visual stimulus—gives a more objective, measurable insight into cognitive and attentional processes involved in, for instance, strategy use or problem solving, without concurrently influencing the process (e.g., Green et al., 2007; Merkley and Ansari, 2010; Huber et al., 2014a). Recording eye movements may therefore be a potential source for capturing thought processes during reasoning and strategy activity. More specifically, and especially with respect to visualizations, it might provide insight into which pieces of information were generally taken into account by a participant and which were not. Thus, eye tracking can be used as a window into cognitive processes that may not be consciously accessible to the participant or apparent to the researcher by task performance (Stephen et al., 2009). Of course, brain-imaging techniques could be a promising additional source of information for combining with techniques like eye tracking within the near future (e.g., see Marian et al., 2003).

Important correlates for cognitive processes during task processing gained by eye tracking are different quantitative and qualitative measures with respect to spatial and temporal features of eye movements that deliver information on eye fixations and saccades. *Fixations* represent the maintaining of the visual gaze on a certain location in the visual field, while fast eye movements from one location to another are called *saccades*. The resulting sequence of fixations and saccades is called a *scan path*, and *dwell time* is the totalized time of all fixations on a given area. In addition, colored *heat maps* aggregate scan paths across different participants, thereby helping researchers to better visualize the relative occurrence of certain scan paths (e.g., see Holmqvist et al., 2011, or Figure 7–10).

Eye movements have already been a valuable tool for investigating a number of cognitive domains, including reading (Verschaffel et al., 1992; Meseguer et al., 2002), visual search (Ho et al., 2001), chess (Charness et al., 2001), and problem solving (Epelboim and Suppes, 2001; Knoblich et al., 2001; Thomas and Lleras, 2007). Meanwhile, eye tracking is also being used increasingly within *educational research* (e.g., van Gog and Scheiter, 2010). With respect to *mathematics education*, there are a number of studies that have applied eye movements for innovative findings, for instance regarding arithmetic word problems (e.g., De Corte et al., 1990; Verschaffel et al., 1992; Hegarty et al., 1995), strategies in solving mental addition problems (Verschaffel et al., 1994; Green et al., 2007), fraction comparison (Huber et al., 2014b; Ischebeck et al., 2015; Obersteiner and Tumpek, 2016), number-line estimation strategies (Schneider et al., 2008; Heine et al., 2010; Sullivan et al., 2011), concepts of angles (Schick, 2012), and equation solving (Susac et al., 2014).

Notwithstanding, Verschaffel et al. (2016) point out that “it is remarkable how little researchers in mathematics education have made use of eye tracking so far, particularly for the identification of strategies” (p. 388).

### Eye Tracking With Tree Diagrams and 2 × 2 Tables

Only a very few studies have analyzed eye movements during the processing of statistical visualizations like tree diagrams or 2 × 2 tables (especially with respect to Bayesian reasoning tasks), although the method seems well suited to investigating cognitive processes in this domain. In the following, we will describe three relevant eye-tracking studies that deal with at least one of the following aspects: (1) Bayesian reasoning situations, (2) tree diagrams or 2 × 2 tables, and (3) information formats (probabilities and frequencies).

Cohen and Staub (2015) examined wrong strategies in Bayesian reasoning based on purely textual statistical information provided in probabilities. They found that several participants consistently used only one of the three probabilities given in a typical Bayesian reasoning problem (see the respective errors in Table 1, e.g., joint occurrence or Fisherian) while other participants used an additive combination of four of the probabilities presented in the tasks (e.g., evidence-only). However, Cohen and Staub (2015) examined only probability versions (but no frequency versions) and did not investigate visualizations in their study.

Lehner and Reiss (2018) analyzed eye movements regarding 2 × 2 tables with absolute numbers (without displaying marginal distributions). However, they did not ask their participants (students) for probabilities or natural frequencies, but rather for decisions (e.g., “Persons of which sex should be asked if...?”; the absolute numbers of female and male people from two countries were given in the corresponding 2 × 2 table). To answer the implemented questions, students had to focus on one or a combination of two, three or all of the four cells of the visualization. Interestingly, the authors found that the students’ gaze durations on single cells differed considerably, with the upper left cell viewed for the most amount of time and the lower right cell for the least amount of time. Moreover, students who were able to solve all of the twelve items with the correct strategy directed their gaze at the lower right cell for a longer period of time than the other participants did. In contrast, students who only solved easier one- or two-cell problems focused for a longer duration on the left column of the table. The authors drew a clear connection between eye movements and (more complex) decision strategies with respect to 2 × 2 tables (Lehner and Reiss, 2018). This research, however, was exclusively focused on 2 × 2 tables containing absolute frequencies and thus tree diagrams or different information formats were not addressed. Furthermore, since no Bayesian reasoning tasks were implemented, the findings cannot be related to Table 1 of this paper.

Finally, Reani et al. (2017) did indeed investigate the effect of the use of different visualizations with regard to Bayesian reasoning problems. With eye tracking they examined visualizations that were presented in addition to text versions, namely tree diagrams (with frequencies), Euler diagrams (as in Figure 2, but with frequencies in the segments of the circles), and icon arrays (without any numerical information). The goal of their study was not primarily to examine whether visualizations facilitate understanding but how students use the presented information. Their eye-tracking data showed that, in line with Lehner and Reiss (2018), participants who answered the presented tasks correctly looked at the stimuli almost twice as long as participants who answered the tasks incorrectly. Regarding frequency trees, they could show that participants looked more intently at information *A* (=total population) than did participants who were presented with a Euler diagram. Conversely, although the performances were identical, regardless of which visualization was used, persons who were shown a Euler diagram viewed information *F* more frequently than persons using a tree diagram (see Table 1). However, Reani et al. (2017) analyzed students’ eye movements only with respect to frequency-based visualizations. This is relevant to note since in secondary school and university, probability format (instead of frequency format) is usually applied, which is much more at risk for possible errors. Yet only by explicitly investigating 2 × 2 tables and tree diagrams with probabilities can one shed light on the seeming discrepancy between the prominent use and, at the same time, the bad performance attributable to probabilistic visualizations (Binder et al., 2015).

Since (German) students are taught statistics based on 2 × 2 tables and tree diagrams, an eye-tracking analysis systematically comparing both visualizations would seem to be a good source of information that could possibly offer insight regarding underlying cognitive processes (including those that result in errors). As statistics (unfortunately) is usually taught almost exclusively based on probabilities and with probability visualizations, a systematic variation of information format within both visualizations is needed in order to explain the benefit of the format change with respect to these two widely used visualizations.

### Present Approach and Research Questions

**FIGURE 4:**
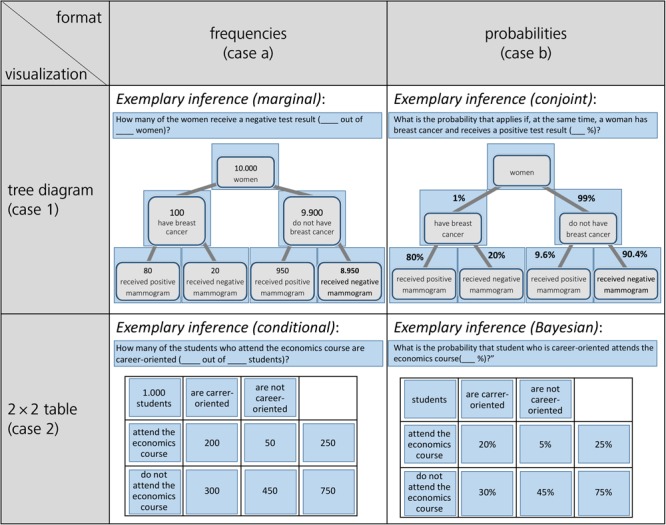
Stimuli for the mammography context and for the economics context (blue-colored AOIs only were included afterwards for the analyses).

The present study provides an empirical basis for interpreting eye movements in terms of strategy use concerning statistical situations containing two binary uncertain events. In our approach, we displayed visualizations (tree diagram vs. 2 × 2 table) of such situations. Instead of presenting a complete textual wording, only the requested inferences were shown (above the visualization). On each new screen displaying a certain task in our computer-based experiment, the information format in the visualization changed from probability to frequency (and vice versa), and the requested inference presented above switched between probability and frequency versions accordingly (see Figure 4 for examples of the final stimuli implemented). In doing so, we examined the strategies of students when they are solving statistical tasks—from easy questions asking for marginal inferences to Bayesian tasks asking for “inverted” conditional inferences (see section Stimuli and Design)—in two different contexts (i.e., mammography context and economics context) by the method of eye tracking, resulting in 20 inferences per participant (see Table 2 for the design). We investigated how participants looked over those visualizations that comprised the relevant statistical information while answering the questions (within a given time limit).

**Table 2:** Design of the experiment (including 20 resulting inferences per participant).

*N* = 24 students		Factor 1: visualization type	
		Tree diagram (context: mammography problem)	2 × 2 table (context: economics problem)
**Factor 2: information format**	Probabilities	•1 marginal •1 conjoint •1 conditional •2 Bayesian	•1 marginal •1 conjoint •1 conditional •2 Bayesian
	Frequencies	•1 marginal •1 conjoint •1 conditional •2 Bayesian	•1 marginal •1 conjoint •1 conditional •2 Bayesian

Our research questions are:

Research question 1:

Which (correct or erroneous) strategies (dependent on visualization type, format, and inference type) used by participants can be detected with the method of eye tracking, and how well can this method predict final performance (i.e., correct or incorrect answer)?

Research question 2:

What can we learn by eye-tracking data about errors made especially in Bayesian reasoning tasks (based on widely applied visualization tools)?

With the first research question (RQ1), we solely want to describe participants’ strategies with “classic” quantitative descriptives such as means of solution rates and error types, and compare these results with corresponding heat maps (obtained by scan paths). Thus, in RQ1, we primarily want to check how validly, reliably, and objectively the method of eye tracking can predict the correctness or error type as documented by the purely numerical answer that participants provide as their solution to the task. Since solution strategies and errors are easier to identify with “simple” inferences, we here start with scan paths of non-Bayesian inferences [i.e., marginal, (non-inverted) conditional, and conjoint] regarding RQ1. If scan paths prove to be a valid indicator of participants’ reasoning strategies in accordance with RQ1, this method can be used in the second research question (RQ2) to shed light on (more complicated) Bayesian inferences. Since the effects of visualization and information format have the highest relevance concerning these notoriously difficult problems, in RQ2 we try to explain by eye-tracking data the benefits and problems inherent in both visualizations considering both formats, especially concerning Bayesian inferences.

According to the results of the studies explicated (see section Eye Tracking With Tree Diagrams and 2 × 2 Tables), we expect to find a clear connection between eye movements and certain strategies (see Lehner and Reiss, 2018), which can be found in corresponding spatial and temporal measures. We furthermore expect tree diagrams to be more adequate for some inference types (e.g., conditional probabilities), which might find expression in higher solution rates. Of course, we also expect a replication of the natural frequency effect. With respect to Reani et al. (2017), we expect to find, for instance, that students focus more on areas that are relevant for answering the corresponding questions as compared to other areas (this should apply equally to both information formats), resulting in a higher dwell time and more fixations.

## Materials and Methods

### Participants

A total of 31 adults, all with normal or corrected-to-normal vision, were recruited as a sample for the experiment. Four of these participants had been tested in a pilot study (their eye-tracking data were not included in the present analysis), and the data of three more participants had to be excluded due to their glasses or technical problems. Thus, *N* = 24 participants (16 female, 8 male) were included in the final analyses. Their mean age was 22.3 (1.6) years, and they ranged from 19 to 26 years of age. The participants were a convenience sample consisting of students from various disciplines at the University of Regensburg (Bavaria, Germany) who were recruited by acquaintance or recommendation. All participants gave their written informed consent and were paid 10 Euro as a representation allowance. While six participants had some unspecific experience with university mathematics due to their studies, the others had only basic mathematical knowledge, and in particular no deeper prior knowledge about (un)conditional probabilities or Bayesian reasoning. Due to their high school education, however, all students were familiar with 2 × 2 tables and tree diagrams containing probabilities, and with 2 × 2 tables containing absolute frequencies, but not with tree diagrams containing frequencies in their nodes (e.g., Binder et al., 2015; Weber et al., 2018).

### Eye-Tracking Device

Participants sat in front of a 19-inch computer monitor (with a screen refresh rate of 100 Hz and a resolution of 1280 × 1024 px) at a viewing distance of 70 ± 10 cm. The screen was connected to a remote eye-tracker (iView XRemote RED 250 mobile by SMI) with a sampling rate of 250 Hz. Throughout each trial, the spatial position of each of the observers’ eyes (“smart binocular”) was sampled running in pupil and corneal reflection mode, resulting in an average spatial accuracy of 0.15°. Participants were asked not to make too many head or body movements, but no device restricted them from moving. Eye movements were calibrated with a five-point, full-screen calibration, both before the experiment began and after a short pause in the middle of the experiment.

### Stimuli and Design

Participants were presented two different statistical situations both involving two binary events, namely the *mammography context* and an *economics context* (the latter adapted from Ajzen, 1977; for both contexts, see also Binder et al., 2015). In Figure 4, all four combinations of information format and visualization type are displayed (with an exemplary inference; further inferences can be seen in Table 3). For each of these two contexts, participants were first asked six non-Bayesian statistical questions—two *marginal*, two (non-inverted) *conditional*, and two *conjoint* inferences, respectively)—in randomized order. After that, they had to answer four (again randomized) *Bayesian* questions in each context, thus resulting in 20 (=2·10) inferences per participant altogether (for the design of the study see Table 2; for the implemented infernces see Table 3; examples of complete stimuli can be seen in Figure 4).

**Table 3:** Categorization of the four possible inference types (Factor 3) for both contexts.

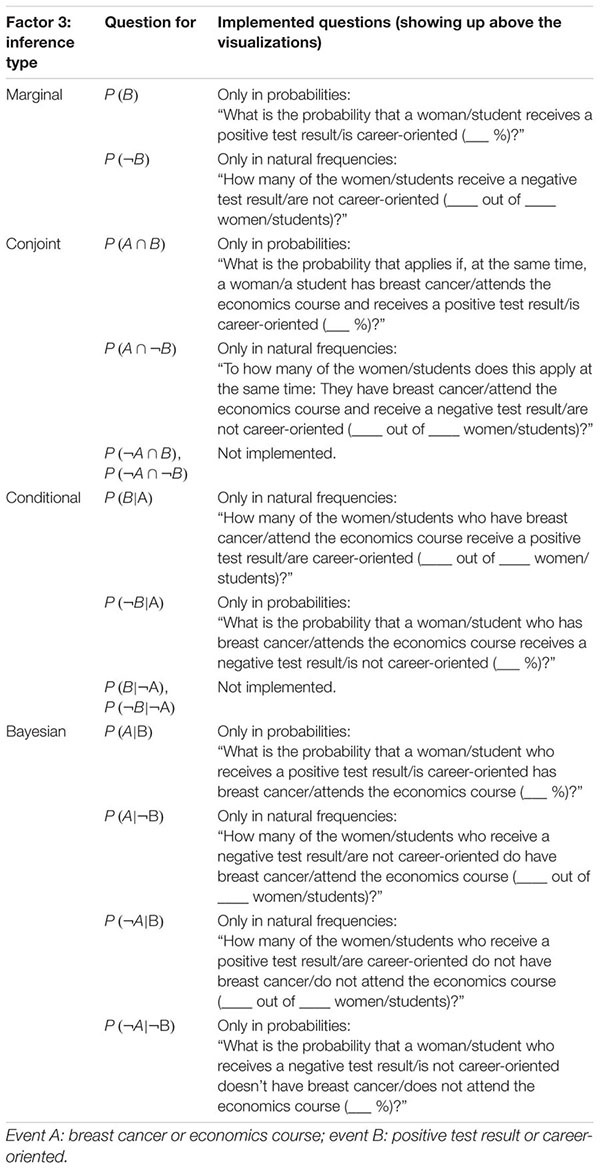

During the administration of each situation (mammography or economics), a large projection of the visualization was shown, with the respective requested inference displayed above the projected image, one after the other. Statistical information on both contexts was given only by this visualization, that is, without additional textual information aside from the question above. To be clear, since both frequency visualizations contain *absolute frequencies*, the term *natural frequencies* strictly speaking refers to the *question format* and not the *information format*. However, absolute frequencies from both visualization types can easily be combined to natural frequencies.

In order to allow familiarization with not only a certain context but also with a specific visualization type, participants always saw a tree diagram for the first ten inferences in the mammography context (factor 1: visualization type). The respective information format within the tree diagram, however, varied randomly, that is, five inferences based on a probability tree and five on a frequency tree (factor 2: information format). Afterward, the same procedure was applied for the ten varying inferences (factor 3: inference type) in the economics context, all of which were based on 2 × 2 tables (again, with a randomly varied information format).

In the following, we refer to non-inverted conditional probabilities simply as “conditional probabilities” and to inverted Bayesian conditional probabilities simply as “Bayesian probabilities.” The difference between both types of conditional probabilities (and the respective frequencies) as expressed by our visualizations is explained in issue 5 of section Number-Based Visualizations: 2 × 2 Tables and Tree Diagrams.

• Factor 1: Visualization type: 2 × 2 table (context: mammography problem) vs. tree diagram (context: economics problem)
• Factor 2: Format of statistical information: probabilities vs. absolute frequencies (or natural frequencies in the corresponding question)
• Factor 3: Inference type: marginal vs. conditional vs. conjoint vs. Bayesian (2×).

In Table 2, the design is illustrated. Since 24 students participated in the experiment, 480 (=24⋅20) inferences were made in total, of which 192 (=24⋅8) were Bayesian inferences. The concrete formulations of the four different types of inferences (displayed above the visualizations) can be found in Table 3.

Thus, from all 16 possible questions (see section Statistical Situations Based on Two Binary Events), we posed 10 questions in each context. Therefore, only two out of four conjoint inferences and two out of four non-inverted conditional inferences are missing (see Table 3), while the also-missing base rates *P(A)* and *P(¬A)* (unconditional probabilities) were posed as sample questions in the introduction to illustrate the procedure.

### Procedure

After a verbal introduction to the experiment that would follow, the procedure began with a short visual introduction [component no. (1), see Table 4]; in order to make participants familiar with the device, several nature pictures were shown on the screen (2).

**Table 4:** Procedure of the experiment.

Part of experiment	Component (no.)
Introduction	(1) Welcome and introduction.
	(2) Six nature pictures for familiarization with the screen.
Part 1 (visualization: tree diagrams;	(3) Calibration.
context: mammography)	(4) Problem introduction (incl. related narrative) and two example inferences.
	(5) Six non-Bayesian inferences.
	(6) Four Bayesian inferences.
Short pause	(7) /
Part 2 (visualization: 2 × 2 tables; context: economics)	(8) Sequence of components (3)–(6) once again.

*The wordings of each task can be found in Table 3.*

In the first part of the experiment (mammography problem with tree diagrams), initial calibration using cornea reflex was conducted (3). If measurement inaccuracy lay below 0.5° in each direction, the experimental procedure itself began, for which we asked participants to avoid head movements as much as possible. Participants were asked to answer as correctly and as quickly as possible. A time limit of 30 s for each inference was implemented to avoid continuing unspecific, non-target-orientated eye movements.

In both parts of the experiment, the problem contexts were introduced with the help of a short related narrative (e.g., “Imagine you are a reporter from a women’s magazine and you want to write an article about breast cancer. You investigate the tests that are conducted in a routine screening in order to detect breast cancer. The following visualization illustrates the situation.”). Then, after participants viewed the situation, they were given two practice trials (4) in order to further familiarize them with the context and both formats (probabilities and frequencies). Both example tasks asked for simple unconditional inferences (i.e., *P(A)* and *P(¬A)* with A being the event “breast cancer” or, in part 2, “economics course”), with one referring to probabilities and the other to frequencies (correct solutions to each were shown afterward). After that, six non-Bayesian inferences followed in random order (5). These six tasks represented a balanced mixture of all possible non-Bayesian tasks (see Tables 2, 3) with respect to format (3 × probabilities, 3 × natural frequencies) and inference type (2 × marginal, 2 × conjoint, and 2 × conditional). If, for instance, one task was given in frequencies [e.g., *P(B|A)*], the other question of the same inference type [P(¬B|A)] was posed in probabilities (see Table 3). At the end of part 1, four Bayesian tasks were presented to the participants (6). While two of the four Bayesian questions [P(A|B),P(¬A|B),P(A|¬B) or P(¬A|¬B)] were asked in probabilities, the other two were asked in natural frequencies. Because Bayesian tasks were presented at the end of each part, participants at this stage were already familiar with the context. Thus by this design, purposeless and merely orientating eye movements should have been avoided at least regarding the four final Bayesian inferences in each context. Whenever the format of questions changed the information format in the tree diagram changed correspondingly.

After a short pause (7), the second part of the experiment (8) was conducted parallel to the first part (a calibration was again conducted beforehand). Regarding the inferences concerning the economics context (all ten based on 2 × 2 tables), each participant received the corresponding inference types again systematically varied (see Tables 2, 3).

Participants were assessed individually in a dimly lit room at the University of Regensburg and were asked to speak loudly and communicate their solutions as quickly and as correctly as possible. When they clicked on the F11-key (or when 30 s ran out), the visualization was no longer visible on the screen, but a fixation cross was shown in the middle of the screen; participants then had to immediately state their answer. The experimenter noted down these verbal responses. No feedback was given to the students during the experimental trials. In order to proceed with the next task, participants were required to click the F11-key on the keyboard once again. It was not necessary to use any other key or the computer mouse. In sum, the whole procedure (including introduction, calibrations, pause, etc.) took about 30–40 min.

With respect to traditional coding, a response was classified as a correct answer if either the exact probability or frequency solution was provided or if the indicated probability answer lay within a one percent interval around the correct answer. For instance, in the mammography problem the correct solution to one of the four Bayesian questions is 7.8%, meaning that answers between 7 and 8% were classified as correct (see also Gigerenzer and Hoffrage, 1995).

### Data Analysis

While stimuli were presented with the software “Experiment Center 3.0,” data analysis of eye movements was conducted using “Suite BeGaze 3.1” (both provided by SMI). To analyze the eye movements, we defined three kinds of “areas of interest” (AOIs) for each screen displaying a task: requested inference (above), concrete information in the visualization, and surrounding white space. Figure 4 displays four sample (out of 20 different) questions (plus AOIs), one for each visualization × format type. (The AOIs do not belong to the stimuli but were only used for analyses.) Please remind that for each of the four visualizations, five inferences were implemented.

More specifically, the AOIs were fitted around the relevant parts of the screen as follows: With respect to the case of tree diagrams with frequencies (see case 1a in Figure 4), both the event and the numerical information were given within the *nodes* of the tree diagram. Here, each of the seven (rectangular) nodes was covered by an equal-sized AOI (each time comprising both number and name of event). In the case of tree diagrams with probabilities (case 1b), numerical information was depicted alongside the *branches* of the diagram; therefore, respective AOIs covered not only the seven nodes (containing the event) but also included the corresponding parts of the branches (containing the respective probability). These AOIs were again equal-sized. In addition, in both cases, the respective inference at the very top of the screen was also covered by an AOI (which was necessarily bigger than the others were). Taken together, eight AOIs covered the whole screen while the rest of the screen was interpreted as a separate area (“whitespace”) representing no information. In the case of 2 × 2 tables with either frequencies or probabilities, respectively, the cells themselves were identified as AOIs for both frequencies and probabilities (cases 2a and 2b). Note that regarding 2 × 2 tables in which the name of the event and the corresponding number are not as close to each other as they are in tree diagrams, the four cells containing the events (“attend the economics course,” “not attend the economics course,” “are career-oriented,” and “are not career-oriented”) were also covered by an additional AOI. In total, this procedure led to eleven equal-sized AOIs for the 2 × 2 table itself, one additional (bigger) AOI for the requested inference, and the remaining whitespace.

## Results

### Research Question 1

**FIGURE 5:**
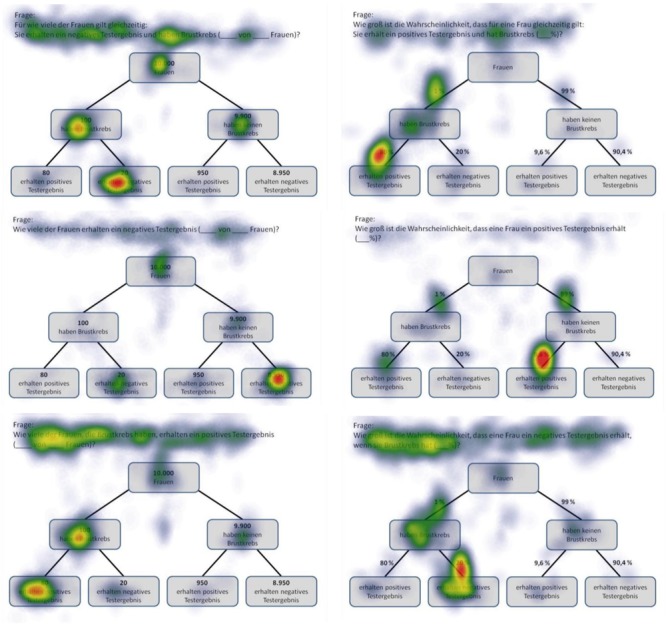
Heat maps of tree diagrams provided with frequencies (left) or with probabilities (right) regarding the following six inferences (from up to below): marginal probabilities, conjoint probabilities, and (non-inverted) conditional probabilities (each only for participants with correct solutions).

**FIGURE 6:**
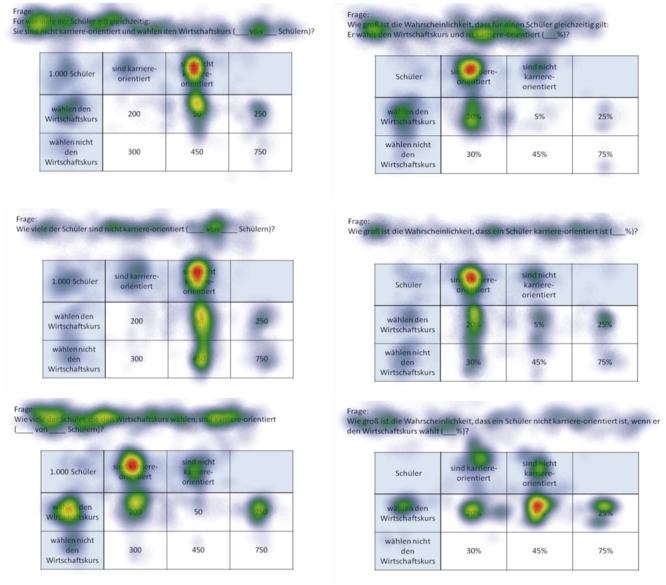
Heat maps of 2 × 2 tables provided with frequencies (left) or with probabilities (right) regarding the following six inferences (from up to below): marginal probabilities, conjoint probabilities, and (non-inverted) conditional probabilities (each only for participants with correct solutions).

Regarding the first research question (RQ1)—“Which (correct or erroneous) strategies (dependent on visualization type, format, and inference type) used by participants can be detected with the method of eye tracking, and how well can this method predict final performance (i.e., correct or incorrect answer)?”—we aim at mapping “classic” quantitative statistics on solution and error rates with the corresponding eye-tracking evidence. For doing so, we first discuss solution rates and errors (Table 5) that are just based on participants’ spoken answers and thus were detectable without eye tracking. Afterward, we report reaction times as well as heat maps regarding participants’ scan paths of correct answers (Figure 5, 6). Finally, we display the quantitative eye-tracking measures such as dwell time and number of fixations (this time across all participants irrespective of correctness of their answers) for the single AOIs (e.g., *A, B, C*, etc., and *b, c, d*, etc.; see Tables 6, 7).

**Table 5:** “Classic” descriptives on all inferences.

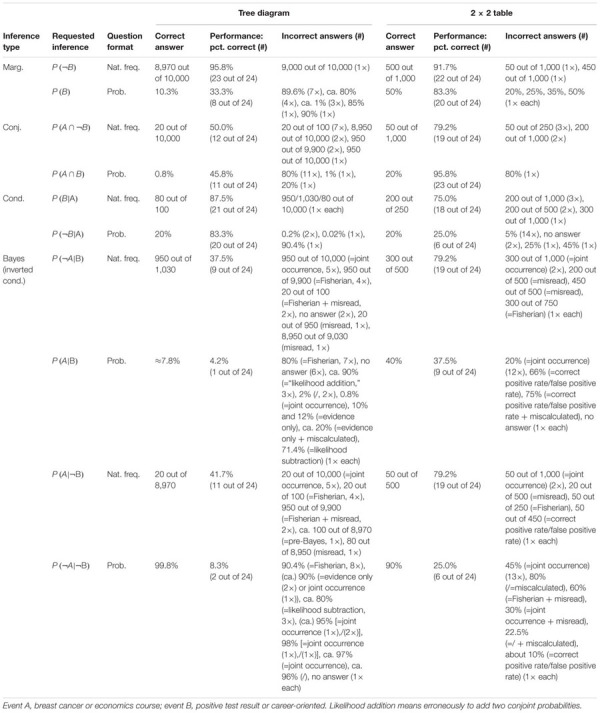

**Table 6:** Quantitative performance indicators regarding AOIs in tree diagrams (mammography context).

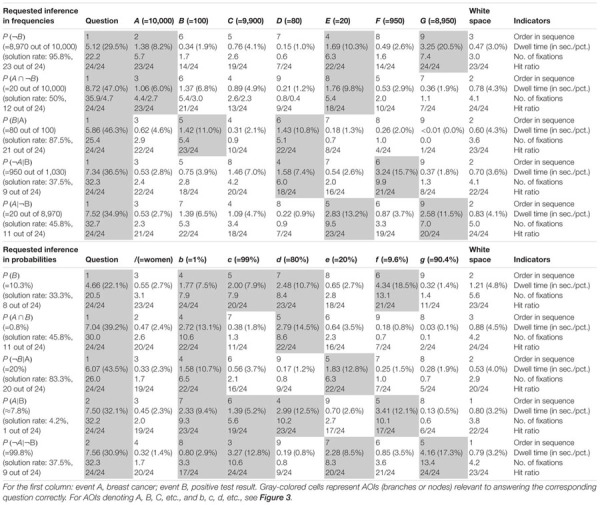

**Table 7:** Quantitative performance indicators regarding AOIs in 2 × 2 tables (economics context).

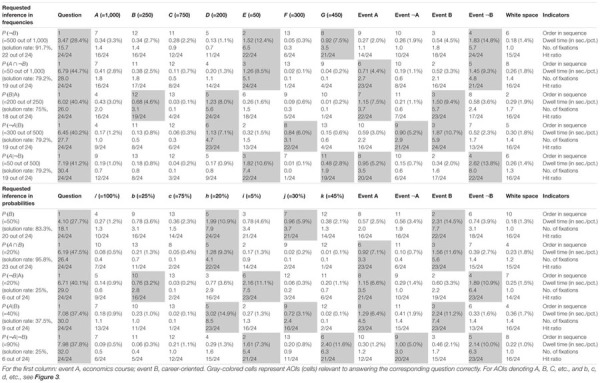

#### Solution Rates and Errors

Although solution rates are clearly not at the center of the present investigation, they are obviously affected by (correct or incorrect) strategies utilized. Table 5 presents an overview of solution rates and the absolute frequencies of specific errors for each of the 20 inferences made by the participants. Solution rates vary substantially, ranging from 4.2 to 95.8% across all conditions.

First, in comparing both *visualization types* (factor 1: tree diagram vs. 2 × 2 table), the considerably different solution rates for structurally identical questions—albeit presented with different contexts—immediately catch the eye. Interesting as that is, however, one must keep in mind when comparing quantitative results between both visualization types that the visualization was not randomized in the current study, since the “mammography trees” preceded the “economics 2 × 2 tables” (see Procedure) because the study initially focused on tree diagrams. Thus learning effects might in fact occur. Nonetheless, 2 × 2 tables proved to be more helpful for “marginal” inferences [P(B), P(¬B)], although only for probabilities (tree: 33.3%; 2 × 2: 83.3%) and not for frequencies (tree: 95.8%; 2 × 2: 91.7%). Questions asking for conjunctions [P(A ∩ B), P(A ∩¬B)] were also answered at a higher rate of error when accompanied by tree diagrams (freq.: 50.0%; prob.: 45.8%) than they were when accompanied by 2 × 2 tables (freq.: 79.2%; prob.: 95.8%). This is in line with theory since conjunctions only have to be read of the screen in 2 × 2 tables (see section Number-Based Visualizations: 2 × 2 Tables and Tree Diagrams). The opposite applies when it comes to (non-inverted) conditional probabilities [P(B|A), P(¬B|¬A)], which were answered with a lower rate of error when accompanied by tree diagrams (freq.: 87.5%; prob.: 83.3%) rather than by 2 × 2 tables (freq.: 75.0%; prob.: 25.0%). Referring to Bayesian inferences (i.e., inverted conditional probabilities), the use of 2 × 2 tables produced either similar or better results than did tree diagrams in relation to all four cases [P(A|B), P(¬A|B), P(A|¬B), P(¬A|¬B)].

Second, regarding *information format* (factor 2: probabilities vs. frequencies), solution rates based on frequencies exceeded those based on probabilities (with one exception) when comparing corresponding questions within tree diagrams (e.g., marginal inferences: freq.: 95.8%; prob.: 33.3%; conjoint inferences: freq.: 50.0%; prob.: 45.8%; conditional inferences: freq.: 87.5%; prob.: 83.3%). The same holds true for the average solution rates of both Bayesian inferences (freq.: 39.6%; prob.: 6.3%). Regarding 2 × 2 tables, similar tendencies were found (marginal inference with freq.: 91.7%; with prob.: 83.3%; conditional inference with freq.: 75.0%; with prob.: 25.0%), except, expectedly, in the case of conjunctions (freq.: 79.2%; prob.: 95.8%). In addition, participants more often solved the two Bayesian tasks correctly in frequency versions than in probability versions (freq.: 79.2 and 79.2%; prob.: 37.5 and 25.0%). When seen in comparison, visualizations presented with frequencies proved to be more easily understandable than those presented with probabilities.

Third, when it comes to different *inference types* (factor 3: marginal vs. conditional vs. conjoint vs. Bayesian), Bayesian tasks, as expected, turned out to be most difficult to solve (39.6% on average across all versions). In probability versions of Bayesian tasks, not only was performance in general relatively low (tree: 6.3%; 2 × 2: 31.3%), but also the kinds of errors that appeared were wide-ranging (see Table 5; we will return to the Bayesian inferences in RQ2). In contrast, solution rates of marginal, conjoint, or conditional inferences (across visualization and format: 76.0, 67.7, or 69.8%, respectively) turned out to be substantially higher meaning that these three kinds of inferences are similarly difficult to solve.

Moreover (and pertinent to the focus of the present investigation), Table 5 exhibits some interesting accumulations of mistakes: Concerning tree diagrams, for instance, some errors regarding non-Bayesian inferences were made by about a third (or more) of all participants [P(A ∩¬B): “20 out of 100” (7×) instead of “20 out of 1,000”; P(A ∩ B): “80%” (11×) instead of “0.8%”; P(B): “89.6%” (7×) instead of “10.3%”]. With Bayesian tasks, participants’ wrong answers naturally piled up all the more [e.g., P(A|B): “80%” (=Fisherian) (7×) instead of “0.83%”; P(¬A|¬B): “90.4%” (=Fisherian) (8×) instead of “99.8%”]. Second, and very similarly, wrong answers regarding inferences based on 2 × 2 tables indicate common deficient strategies. Most often by far, the (non-Bayesian) conditional probability P(¬B|¬A) produced a great number of identical wrong answers [e.g., “5%” (14×) instead of “20%”]. The same holds true for the Bayesian inferences in which two wrong answers in particular (both conforming to joint occurrence and both based on probabilities) appeared to be very tempting [P(A|B): “20%” (12×) instead of “40%”; P(¬A|¬B): “45%” (13×) instead of “90%,” see Table 5]. In all of these cases, analysis of scan paths might reveal a deeper understanding of the specific errors (for details see below).

#### Reaction Times

Interestingly, the average time it took for participants to reach a solution was not remarkably different for correct or incorrect solutions (in contrast to Reani et al., 2017). In fact, we found differential effects with respect to both visualization types. For instance, regarding the four Bayesian inferences based on tree diagrams, participants who solved the tasks correctly took slightly more time than those who did not [Bayesian inferences with tree diagrams: *M*(*SD*)_correct_ = 23.57(5.78) sec. vs. *M*(*SD*)_incorrect_ = 22.06(7.05) sec.; small effect of *d* = 0.23 according to Cohen, 1992]. In contrast, with respect to the corresponding four Bayesian inferences based on 2 × 2 tables, the opposite is true: 2 × 2 tables were looked at for a longer period of time by participants who came up with incorrect solutions than by those who gave correct answers [Bayesian inferences with 2 × 2 tables: *M*(*SD*)_correct_ = 17.31(5.78) sec. as compared to *M*(*SD*)_incorrect_ = 20.03(7.69) sec., *d* = -0.40] (also see Binder et al., unpublished).

#### Cognitive Strategies Heat Maps Displaying Correct Answers

Before we begin our analysis, we should mention a qualitative aspect that we immediately noticed about participants’ scan paths: Participants tended to look back to the requested inference after initially having looked forward to the inference, and after that to the visualization. It seems as if they wanted to make sure that they had understood the requested inference correctly (see also Tables 6, 7). This occurred even more frequently when the question was either difficult (i.e., low solution rate) or the person subsequently answered the question wrongly.

Heat maps can present the scan paths of, for instance, participants who solved the tasks correctly. In Figure 5, such heat maps regarding all six non-Bayesian inferences based on tree diagrams are presented. Corresponding heat maps regarding Bayesian inferences (based on tree diagrams or 2 × 2 tables) are displayed in Supplementary Material. These colored maps can serve as an indicator for the validity, reliability, and objectivity of the method in general: As can be seen in Figure 5, nodes and branches that were relevant for solving the task based on a given tree diagram precisely and distinctly correspond to the areas at which participants looked for the longest period of time. The same holds true with respect to 2 × 2 tables (see Figure 6). Taken together, heat maps indicating the most-viewed areas of a stimulus provide a first clue that participants’ (individual) viewing areas correspond to their (individual) viewing strategies.

**FIGURE 7:**
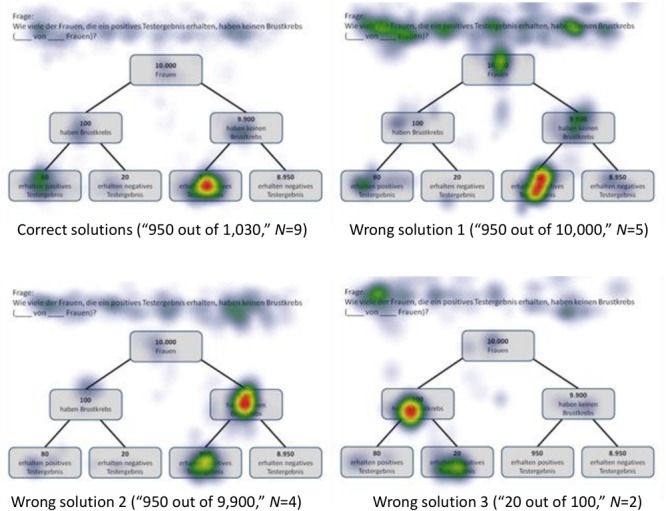
Heat maps regarding the Bayesian inference P(¬A|B) with a tree diagram (mammography context) with frequencies.

**FIGURE 8:**
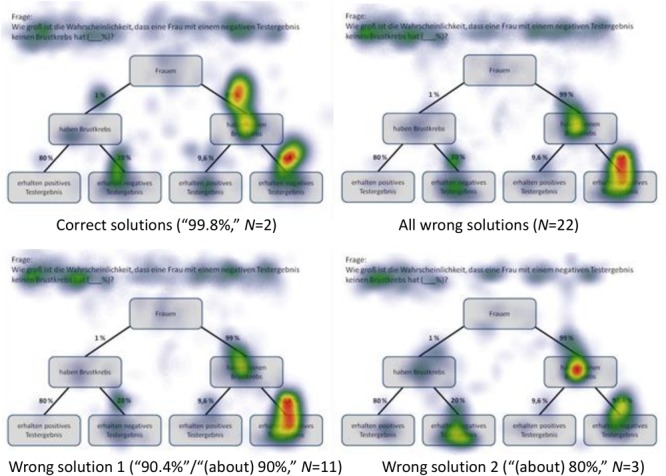
Heat maps regarding the Bayesian inference P(¬A|¬B) with a tree diagram (mammography context) with probabilities.

**FIGURE 9:**
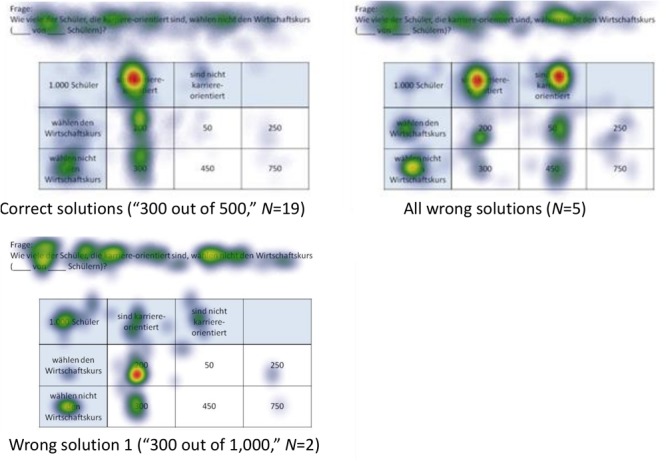
Heat maps regarding the inference P(¬A|B) with a 2 × 2 table (economics context) with frequencies.

**FIGURE 10:**
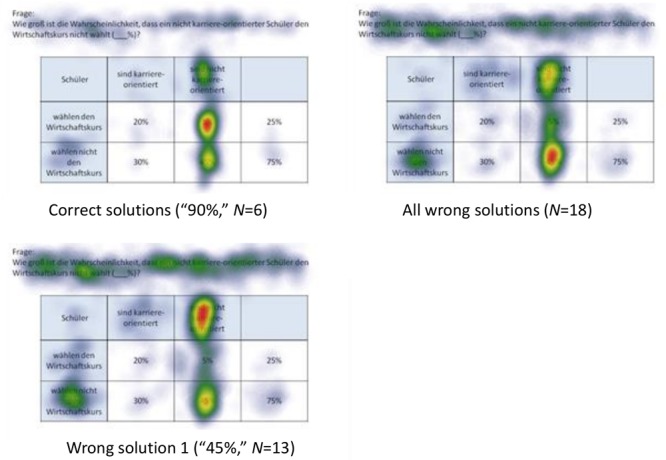
Heat maps regarding the inference P(¬A|¬B) with a 2 × 2 table (economics context) with probabilities.

Because in eye-tracking studies it is not possible to present all qualitative results in detail, only heat maps regarding correct solutions are presented here (see Figure 5, 6 for all implemented non-Bayesian inferences, Figure 7–10 for four sample Bayesian inferences, and Supplementary Material for the other four Bayesian inferences). Since heat maps in general prove to be valid indicators of participants’ focused areas, and because errors are much more relevant concerning Bayesian inferences, we will return to “*Bayesian error* heat maps” in section Research Question 2.

#### Quantitative Eye-Tracking Analyses of AOIs (Across Correct and Wrong Answers)

Quantitative eye-tracking data refer to the single AOIs, as labeled in Figure 3 (*A, B, C*, etc., and *b, c, d*, etc., respectively). The upper halves of Table 6 (mammography context) and Table 7 (economics context) report results regarding nodes or cells of frequency visualizations, and the lower halves those regarding the corresponding AOIs in probability visualizations. Each cell in both tables displays what is known as *performance indicators* that are calculated on average for all participants irrespective of the correctness of their answers, and which are (from top to bottom in each cell) (a) the ordinal number of a certain AOI considered in the sequence (scan paths), (b) the overall dwell time on the respective AOI (in seconds and percentage-wise), (c) the total number of fixations on this AOI, and (d) the hit ratio (i.e., by how many participants the AOI was viewed). In both tables, gray-colored cells represent AOIs that were relevant to answering the corresponding questions, while the other cells were not relevant. For instance, to compute P(¬B) (correct answer: “8,970 out of 10,000”), one has to add the numbers in the AOIs *E* (“20”) and *G* (“8,950”) and put the sum in relation to *A* (“10,000”). Because of the small sample size, in the following we present no inference measures (i.e., *p*-values) in favor of qualitative interpretations.

The *order in sequence* is a condensed measure representing the order in which participants scanned the visualization. Considering all of these numbers within a scan path, this measure corresponds to what participants’ averaged scan paths look like chronologically. Quite irrespective of whether an AOI is of relevance or not to answer the corresponding question, both visualization types were tendentially viewed for the first time from top to bottom and from left to right [e.g., see P(B|A)]. To be clear, the requested inference is considered first. In tree diagrams, the underlying sample (size) is usually viewed after that (which is the AOI *A* for frequency or the AOI “women” for probability versions), while in 2 × 2 tables, participants usually next looked at event B and the upper cells (which are *D* and *E* for frequency or *h* and *i* for probability visualizations).

The *dwell tim*e represents the time added up of a participant’s fixating on a certain AOI, and therefore is necessarily highly correlated with *number of fixations* (see next paragraph). It is not surprising that the AOI that attracted the most attention by far was the requested inference at the top of the screen. Participants spent between 20% and 50% of their time looking at this area. In more detail, both the percentage of time and the absolute time spent on this instruction were especially high for Bayesian questions [e.g., P(¬A|¬B)] and relatively low for (easier) marginal inferences [e.g., P(¬B)]. This finding indicates that participants needed more time (to grasp and understand the requested inference correctly) the more difficult the inferences were. In addition, AOIs that had to be looked at in order to answer the questions (i.e., gray-colored cells) attracted more attention than those that were irrelevant. With only a few exceptions [e.g., P(¬A|¬B) with tree diagrams], the dwell time in the relevant AOIs (gray-colored cells) for any inference was always higher than the dwell time in the irrelevant AOIs.

The *number of fixations* is simply a total of single fixations that occurred in an AOI. As can be seen with respect to both visualizations, the number of fixations was nearly always highest for the AOIs that contained information that was necessary to answering the corresponding question (gray-colored cells). For instance, answering the conditional probability P(B|A), participants spent at least three fixations on the two relevant AOIs (cells *B* and *D*) and almost completely ignored all others. With only one exception [namely, the AOI *f* for P(¬A|¬B) in the tree diagram with probabilities], the average number of fixations on the relevant AOIs was always higher than the average number on all of the irrelevant AOIs. These results further indicate that participants process the information in the relevant areas more intensively.

The *hit ratio* represents the proportion of (all 24) participants who looked at the respective AOI. While—not surprisingly—all participants in each instance viewed each task’s instructions, some of the irrelevant AOIs were almost completely ignored, which was true especially for the very easy questions [e.g., P(B|A) for tree diagrams or P(A ∩ B) for 2 × 2 tables]. This finding indicates that participants are effectively able to find the relevant information.

In sum, not only heat maps but also performance measures regarding the AOIs (i.e., indicators like order in sequence, dwell time, etc.) obviously provide meaningful evidence of participants’ reasoning processes. Both kinds of measures (see Figure 5, 6 and Tables 6, 7) can not only be matched with solution and error rates (Table 5), but also partly explain erroneous strategies (e.g., Fisherian). This motivates the consideration of these measures with respect to Bayesian inferences in RQ2.

### Research Question 2

In the following, we will analyze how solution strategies in Bayesian tasks as evidenced by heat maps and performance indicators (i.e., dwell time, etc.) are impacted by the varying of the two factors *visualization type* and *format*. To do so, we take the two Bayesian inferences P(¬A|B) and P(¬A|¬B) as sample tasks (A reminder: While performance rates of all Bayesian inferences are summarized in the lower half of Table 5, performance indicators based on the AOIs of all Bayesian inferences can be found in Tables 6, 7). Heat maps of the two chosen Bayesian inferences, P(¬A|B) and P(¬A|¬B) (both for the correct and the most frequent incorrect strategies), are displayed in Figure 7–10, whereas the respective heat maps regarding the two unchosen Bayesian inferences, P(¬A|B) and P(¬A|B), can be found in Supplementary Material. Note that while performance measures of AOIs (Tables 6, 7) again are summarized across all participants’ strategies, the heat maps (Figure 7–10 and Supplementary Material) explicitly distinguish between correct and incorrect answers.

#### *P*(¬*A*|*B*), Based on a Tree Diagram With Frequencies (Mammography Context)

*N* = 9 participants solved the task P(¬A|B), which asked for a Bayesian inference with frequencies [correct solution: “950 out of 1,030” = “950 out of (950+80)” = “*F* out of (*F*+*D*)”]. As might be expected, participants focused mainly on the relevant AOIs (nodes) *D* (“80”) and *F* (“950”) (but also on *A* and *C*; see Figure 7). In doing so, they focused much more on *F* (than on *D*), which is relevant for both the numerator and the denominator during calculation (besides the mere size of the number). This finding is supported by the high values of number of fixations and dwell time in the corresponding AOIs (although all participants are included, not just those with correct answers).

More interestingly, and of relevance for RQ2, with respect to wrong answer 1 (“950 out of 10,000,” *N* = 5), the scan paths are very similar to those evidenced when selecting the correct answer. For obvious reasons, node *A* (“10,000”) was focused on to a greater extent, resulting in a calculation of the “marginal frequency” P(B) (=error “joint occurrence”). In addition, participants focused more on the question provided above the visualization. With respect to wrong answer 2 (“950 out of 9,900,” *N* = 4), participants heavily focused on *C* (“9,900”) in addition to *F*, therefore erroneously calculating the conditional probability P(B|¬A) (=Fisherian). Finally, participants giving incorrect answer 3 (“20 out of 100,” *N* = 2) focused on the corresponding AOIs *E* (“20”) and *B* (“100”), which means that they calculated the “conditional frequency” P(B|A). Obviously, the latter two participants not only executed the wrong calculations, but also misread the question (“receive a negative test result” instead of “receive a positive test result”) (=Fisherian).

#### *P*(¬*A*|¬*B*), Based on a Tree Diagram With Probabilities (Mammography Context)

The question P(¬A|¬B) required a Bayesian inference with probabilities and was solved correctly by only *N* = 2 participants [correct solution: “99.80%” = 99%⋅90.4%/(99%⋅90.4% + 1%⋅20%) = “(*c*⋅*g*)/((*c*⋅*g*) + (*b*⋅*e*))”]. Participants with the correct answer (all answers between 99 and 100% were classified as correct) focused mainly on the relevant AOIs (branches) *c* (“99%”) and *g* (“90.4%”) and on the AOIs *b* and *e* (see Figure 8), which are relevant for both the numerator and the denominator during calculation. This finding is supported by the maximally high hit ratio (24 out of 24 hits each on AOIs *c* and *g*) and also by the quite high values of dwell time and number of fixations in the corresponding AOIs.

The heat map of all wrong answers (*N* = 15) reveals a particular focus on the AOI *g* (“90.4%”), which was also true for the most prominent wrong answer [“90.4%” (*N* = 8) or “(about) 90%” (*N* = 3)]. Obviously, some of these participants thought that they could simply read on the screen the correct answer from AOI *g* (“90.4%”). Alternatively, some others thought that they had to multiply “90.4%” (AOI *g*) by “99%” (AOI *c*) (≈ 90%). In any case, this is why they more or less ignored the (relevant) AOIs *b* and *e*. While the first incorrect answer represents a conditional probability (=Fisherian), the second corresponds to a conjoint probability [=joint occurrence, or the error “evidence only” = (*c*⋅*g*)+(*b*⋅*e*)]. Eye-movement patterns helped to distinguish, for instance, Fisherian from conjoint occurrence errors, even though both mistakes result in nearly the same incorrect answer (e.g., “90.4%” and “ca. 90%,” but also “95%” or “98%”). Regarding wrong answer 2 [“(about) 80%,” *N* = 3], participants’ viewing patterns were quite similar to those of participants who solved the task correctly. Interestingly, as can also be seen in Figure 8, their answer, “80%,” is obviously not due to AOI *d* (“80%”), which they more or less ignored, nor to the subtraction “90.4–9.6%” (=*g*–*f*). Instead, it seems that they calculated “90.4%–20%” (or “99%–20%”) (=likelihood subtraction). Thus, with respect to RQ2, incorrect reasoning strategies could be detected (only with the help of eye-tracking data) that were not obvious in the given wrong answers itself.

#### *P*(¬*A*|*B*), Based on a 2 × 2 Table With Frequencies (Economics Context)

P(¬A|B) asked for a Bayesian inference with frequencies. It was solved by *N* = 19 participants [correct solution: “300 out of 500” = 300 out of (300+200) = “*D* out of (*D*+*F*)”]. Participants focused mainly on the relevant AOIs *D* (“300”) and *F* (“200”), each to a similar extent (see Figure 9). In addition, they also focused on the marginal cells “choose the economics course” (event A) and—to an even greater extent—“is career-oriented” (event B), which also finds expression in, for instance, the dwell time and hit ratio on the corresponding AOIs.

With respect to all wrong answers (*N* = 5), the heat map shows that the marginal cells “choose the economics course” (event A) and (the irrelevant) “not choose the economics course” (event ¬A) were focused on most, both to a very similar extent. However, regarding wrong answer 1 (“300 out of 1,000,” *N* = 2), the corresponding participants’ viewing patterns were somehow similar to those of participants with correct solutions, except that the former focused heavily on *D* (“300”). Also, in contrast to the participants who gave the correct answer, they focused substantially on the marginal cell “1,000 students,” which was part of their answer, thus providing a “marginal frequency” (=joint occurrence).

#### *P*(¬*A*|¬*B*), Based on a 2 × 2 Table With Probabilities (Economics Context)

Only *N* = 6 participants solved the question P(¬A|¬B) correctly, which asked for a Bayesian inference based on a 2 × 2 table provided with probabilities [correct solution: “90%” = 45%/(45%+5%) = “*k*/(*k*+*i*)”]. Participants who gave the correct answer focused mainly on the relevant cells *k* (“45%”) and *i* (“5%”) (see Figure 10). Interestingly, in doing so, they focused much more on *i* (than on *k*), which is relevant only for the calculation of the denominator. This may be because the cell *i* is positioned between the other two relevant cells. They also focus substantially on the marginal cell “are not career-oriented,” which represents the condition ¬B. This finding is supported by the values of number of fixations, dwell time, and hit ratio in the corresponding AOIs (all participants are included).

The heat map of all wrong answers (*N* = 18) reveals a stronger focus on cell *i* (“5%”) in addition to the corresponding marginal cells (“not choose the economics course” and “are not career-oriented”). The same holds true for the most relevant wrong answer (“45%,” *N* = 13): Obviously, these participants thought that they could read the correct answer from the screen in cell *k* (“45%”), which is why they more or less ignored the (relevant) cell *i* (“5%”). In doing so, their answer once again erroneously represents a conjoint probability (=joint occurrence).

In sum, the analysis of scan paths by eye tracking revealed, aside from some instances of apparent misreadings, miscalculations, and undefined mistakes, the following recognized errors that can occur in Bayesian tasks (see Table 8): The errors “joint occurrence” (in sum: 45×) and “Fisherian” (30×) happened by far the most often. While Fisherian occurred more frequently with tree diagrams (27×) than with 2 × 2 tables (3×), the opposite applies for joint occurrence (tree: 15×; 2×2: 30×). This mismatch is especially due to the high number of joint occurrence errors involving 2 × 2 tables with probabilities (26×), but not involving those with frequencies (4×). All of the other cited errors (e.g., “Pre-Bayes,” “likelihood subtraction,” etc.; see Table 1) could be found in the scan paths and the corresponding answers, but in sum, only quite seldom (15×).

**Table 8:** Errors per visualization × question/information format for Bayesian inferences.

Visualization	Format: Frequencies	Probabilities
**Tree diagram**	10× joint occurrence12× Fisherian1× Pre-Bayes(in sum: 23× established errors out of 28 errors)	5× joint occurrence15× Fisherian3× likelihood subtraction3× “likelihood addition”3× evidence only(in sum: 29× out of 45 errors)
**2 × 2 table**	4× joint occurrence2× Fisherian(in sum: 6× out of 10 errors)	26× joint occurrence1× Fisherian3× correct positive rate/false positive rate(in sum: 30× out of 33 errors)

*48 Bayesian inferences per combination.*

## Discussion

### Conclusion

An original feature of this study was the collection of scan paths produced by eye movements during statistical reasoning processes based on tree diagrams and 2 × 2 tables (both provided with probabilities or frequencies). Analyzing students’ viewing strategies for solving statistical tasks proved useful as a valid, detailed, and sensitive indicator of participants’ reasoning strategies (RQ1). These eye movements provided insight into temporal and spatial distributions of attention during the processing of specific visualizations that are widely applied in the teaching of statistics, not only in Germany but also in many other countries. Since the visualizations provided were presented with either probabilities or frequencies, the participants’ solutions also give some hints regarding the benefits and pitfalls (such as provoking particular recognized errors) of different formats in different visualizations. In this way, they call for didactical consequences with respect to the teaching and learning of statistical and especially Bayesian reasoning.

Concerning Bayesian inferences (RQ2), which are intensively examined in cognitive psychology because of their relevance for expert decision-making in various domains, we specifically found the following: Regarding different *visualization types*, tree diagrams clearly elicit more different kinds of errors than do 2 × 2 tables (see Table 8). Viewing patterns (i.e., heat maps) that are essentially a representation of incorrect solutions indicate that 2 × 2 tables especially provoke answers equaling marginal probabilities (or frequencies)—a mistake which is called “joint occurrence” (see Table 1). This is logical insofar as 2 × 2 tables, solely due to their structure, display conjoint probabilities in their central cells, thus very much focusing on these probabilities (or frequencies). Moreover, we found only few more established mistakes (i.e., Fisherian, see Table 8). Tree diagrams, on the other hand, elicit a variety of incorrect calculations for both formats: We most often encountered “joint occurrence” and “Fisherian,” but occasionally “pre-Bayes,” “likelihood subtraction,” and “evidence-only” as well (see Table 8). Thus even though there are obvious benefits of tree diagrams (e.g., see Binder et al., 2015), they more frequently led to different kinds of erroneous calculations in Bayesian questions. One could speculate on whether this is due to their hierarchical structure (contrary to the non-hierarchically structured 2 × 2 tables), which, for example, finds expression in better performances for (non-inverted) conditional inferences for tree diagrams (see Table 5). In addition, eye-tracking patterns (i.e., scan paths and heat maps) also revealed that some mistakes were caused by simple misreading (e.g., oversight of a negation) or miscalculations.

Regarding different *formats*, tasks with frequencies were solved to a substantially larger extent than those with probabilities. This result is also reflected in the briefer period of time required to solve frequency tasks (irrespective of whether correct or incorrect answers are compared). Regarding Bayesian inferences, though most participants identified the relevant AOIs for answering a specific inference (as mirrored by dwell time and hit ratio, see Tables 6, 7), neither information format could inhibit the most relevant errors (especially “joint occurrence” and “Fisherian”). The corresponding scan paths and aggregated heat maps (e.g., see Figure 7–10) support these findings. While participants made only a few different errors in questions posed in natural frequencies, tasks posed in probabilities provoked a greater variety of mistakes, for instance “likelihood addition” (which means erroneously to add two conjoint probabilities) and “evidence only,” in addition to some unspecific errors. It seems as if, in contrast to the probability format, the format of frequencies not only reduces errors in general, but also prevents participants from unusual errors (presumably, since the nodes and the cells can very flexibly be combined to multiple insight-fostering natural frequencies).

With respect to different *inference types*, the solution rates of Bayesian tasks expectedly were lower than those of the other inference types. This result also finds expression in the dwell time that participants spent in looking at the instruction: This quantitative measure was especially high for Bayesian questions (and low for marginal inferences). Moreover, we found that participants considered task-relevant AOIs more important than irrelevant AOIs, irrespective of the requested inference type (which is reflected in a higher hit ratio, dwell time, and number of fixations for relevant AOIs). In detail, regarding Bayesian inferences, some typical erroneous Bayesian calculations (see Table 1) occurred quite often, while we could detect some others only very rarely (see Table 8). Presumably, this finding is due to the given visualizations (rather than mere textual information), which obviously prevents participants from experiencing some (infrequent) misunderstandings.

In sum, and especially with respect to RQ2, the analyses of individual scan paths helped to identify certain strategies, which would not have been possible without eye tracking. For instance, eye tracking helped in interpreting (incorrect) answers that otherwise would have seemed like “nonsense” answers but now could be attributed to misinterpretation, misreading, or miscalculation (see Table 5, e.g., for P(¬A|¬B) with 2 × 2 tables). Moreover, and especially with respect to probability visualizations in Bayesian tasks, eye-movement analyses revealed that different answers sometimes arise from basically the same errors (see, e.g., P(¬A|¬B) with tree diagrams). Conversely, eye tracking helped to distinguish different errors from the same (or very similar) erroneous answers (also see, e.g., P(¬A|¬B) with tree diagrams). Furthermore, eye-tracking data revealed that both visualization types are often considered from top to bottom and from left to right (as indicated by the order of sequence), quite similar to the way in which one usually reads a text. Last but not least, participants viewed the requested inferences for quite a long time (and their gaze often returned to them, especially in the case of Bayesian tasks).

The above-mentioned findings, especially the occurrence of very different error distributions with respect to different visualization types and information formats, lead to the following recommendations with respect to the teaching and learning of Bayesian situations: With the results from all inference types (i.e., marginal, conjoint, conditional, and Bayesian) in mind, visualizations should be taught in a more *integrative* and *contrasting* way. This means that, apart from merely showing the visualization (and grasping the relevant information on its own), the “location” of certain information could be explicitly made obvious, for instance by marking the relevant branches or nodes (see Binder et al., 2018). Furthermore, the location of some probabilities or frequencies could explicitly be compared with the location of the same information in other visualizations in order to contrast the different visualizations and information formats (and thus also their advantages and disadvantages). This might lead to a better understanding of which information tree diagrams and 2 × 2 tables display directly (and where), and which inferences cannot be read off but have to be calculated through combining different numbers. In this way, less mixing up of different inference types should occur. Finally, teachers could emphasize the intelligent reading of visualizations (see Curcio, 1989). For instance, if a conditional probability P(B|A) has to be read or computed from a 2 × 2 table, it is somehow more straightforward to focus on the condition first (i.e., on event A, in our study depicted in the columns), and only after that to focus on the corresponding unconditional event (i.e., on event B) in order to compute the correct probability. In tree diagrams, students have to understand that only one “reading direction” is displayed, and thus only one piece of marginal information can be directly read from the tree. In contrast, in double-tree diagrams (e.g., see Wassner, 2004; Khan et al., 2015) both reading directions are displayed at a glance, which is advantageous for teaching conditional probabilities. In our study, the scan paths of many participants led us to believe that they did not have a deep understanding of how both of the presented visualizations were structured (although they certainly were confronted with them in secondary school).

### Limitations of This Study, and Possible Future Research

Qualitative and quantitative eye-movement data and participants’ accuracy (i.e., solution rates) provide support for distinguishing among (perhaps unconscious) strategies. Nevertheless, it is necessary to acknowledge that strategies here were derived only indirectly through (aggregated) scan paths (i.e., heat maps), accompanied by the participants’ answers. More generally, as it holds true for all eye-tracking studies, it has to be conceded that eye movements and strategy use are by nature related but distinct indicators of thought processes. This is because—similar to gesture—any strategy principally can be performed without the corresponding eye movements as long as the meanings and locations of all the numbers and symbols (e.g., distinct probabilities or frequencies) are known. Future studies, for instance accompanied by retrospective questions to the students intended to help them to figure out their (conscious) strategies, could even more deeply enhance our understanding of participants’ thinking.

Moreover, eye-movement data for strategy identification in the domain of mathematical cognition have some general pitfalls (see Verschaffel, 2014): The “process of solving a mathematical problem typically not only consists of an execution phase, but also of an orientation and (possibly) a verification phase” (see Verschaffel et al., 2016, p. 388). Those phases are experimentally hard to separate from each other. In addition, even if one were able to isolate the execution phase, it “frequently may not consist of the straightforward running of a single well-identifiable strategy” (see Verschaffel et al., 2016, p. 388). Taken together, strategies cannot be derived that easily or incautiously. However, we tried to minimize those concerns by keeping the related narrative and the context constant, only changing the corresponding inference (and the information format in the visualization accordingly).

We further acknowledge the limitation that participants were always shown tasks with tree diagrams first, which were then followed by questions with 2 × 2 tables, maybe resulting in a certain learning trajectory from tasks with tree diagrams to those with 2 × 2 tables. Further confounding variables with respect to a comparison of both contexts (and consequently of both visualization types) were somewhat “easier” numbers, the counterintuitive low base rate [i.e., P(A)], and the context itself that might disadvantage tree diagrams as compared to 2 × 2 tables (see also Siegrist and Keller, 2011, for differences in performance of participants in different contexts). For these reasons, comparisons of solution rates and distribution of various mistakes have to be made very cautiously, which might also affect the heterogeneity of wrong answers to some extent. Furthermore, the number of participants was relatively low—although very small case numbers are actually common in eye-tracking studies due to the complexity of their technical implementation. Since quantitative measures obtained can therefore only be interpreted restrictedly, we refrained from inferential statistics. Due to the different structure of both visualization types (hierarchical vs. non-hierarchical) and the location of statistical information (branches or nodes in tree diagrams vs. cells in 2 × 2 tables), both the numbers and the sizes of areas of interest cannot be kept completely comparable, thus in some ways biasing quantitative measures in different conditions. A potential solution to this problem might be to standardize quantitative measures (e.g., fixations) by dividing their number or length by the size and/or number of the respective AOIs.

For future research, it would be interesting to examine the effect of different textual problem formulations on strategies (e.g., for conjoint probabilities, see Hertwig et al., 2008; for conditional probabilities, see partitive vs. non-partitive formulations in Macchi, 2000), since understanding and strategy use are obviously heavily affected by linguistic competencies. In the mammography problem, the more complicated terminology and/or cognitively taxing scenario could also account for the different effects in the different contexts (e.g., Lesage et al., 2013; Sirota et al., 2014a).

Regarding visual aspects, it would also be interesting to analyze the effect of special characteristics of visualizations on viewing patterns. For instance, instead of presenting “normal” tree diagrams or 2 × 2 tables, one could display visualizations with highlighted branches, nodes, or cells in order to figure out the visualizations’ effect on participants’ eye movements (“signaling principle,” see section Number-Based Visualizations: 2 × 2 Tables and Tree Diagrams). Furthermore, it would be interesting to determine whether and how both resources of information (textual and visual) can be integrated or not (and thus shed more light on the “redundancy principle,” see section Number-Based Visualizations: 2 × 2 Tables and Tree Diagrams).

Last but not least, the expert-novices paradigm promises some new insights, for example with respect to certain patterns of mistakes: In comparing scan paths and strategies of novices with those of experts, one could perhaps make “learning visible” over time.

## Ethics Statement

This study was carried out in accordance with the recommendations of ‘Ethikkommission an der Universität Regensburg’ with written informed consent from all subjects. Students were informed that their participation was voluntary, and anonymity was guaranteed. After the study participants were debriefed.

## Author Contributions

GB, KB, and SK contributed by writing the draft of the manuscript. All authors listed have made substantial, direct, and intellectual contribution to the work and approved it for publication. In addition, H-MK and GB recorded the data.

## Conflict of Interest Statement

The authors declare that the research was conducted in the absence of any commercial or financial relationships that could be construed as a potential conflict of interest.
