# Supplementary material for: An Eye-Tracking Study of Statistical Reasoning With Tree Diagrams and 2 × 2 Tables
Source: Front Psychol. 2019 May 15;10:632. doi: 10.3389/fpsyg.2019.00632 (PMC6530428; doi:10.3389/fpsyg.2019.00632)
Supplement: Supplementary file 1 [file Table_1.DOCX]

Supplementary Material

An eye-tracking study of statistical reasoning with tree diagrams and 2×2 tables

Georg Bruckmaier,^1*^ Karin Binder,^2^ Stefan Krauss,^2^ and Han-Min Kufner^2^

^1^ Department of Secondary Education, University of Education, University of Applied Sciences and Arts Northwestern Switzerland, Brugg, Switzerland

^2^ Mathematics Education, Faculty of Mathematics, University of Regensburg, Regensburg, Germany

*** Correspondence:** Georg Bruckmaier, Department of Secondary Education, University of Education, University of Applied Sciences and Arts Northwestern Switzerland, Brugg, Switzerland

Georg.Bruckmaier@fhnw.ch

**Supplementary table 1.** Problem formulations of the economics problem according to Ajzen (1977) in probabilities and natural frequencies

| **Probabilities** | Imagine you are interested in the question, if career-oriented students are more likely to attend an economics course. Therefore the school psychological service evaluates the correlations of personality characteristics and choice of courses for you. The following information is available:  The probability that a student attends the economics course is 32.5%.  If a student attends the economics course, the probability that he is career oriented is 64%.  If a student does not attend the economics course, the probability that he is still career-oriented is 60%.  **Question:** What is the probability that a student attends the economics course if he is career-oriented?  **Answer:** About 34 % |
| --- | --- |
| **Natural frequencies** | Imagine you are interested in the question, if career-oriented students are more likely to attend an economics course. Therefore the school psychological service evaluates the correlations of personality characteristics and choice of courses for you. The following information is available:  325 out of 1,000 students attend the economics course.  Out of 325 students who attend the economics course, 208 are career-oriented.  Out of 675 students who not attend the economics course, 405 are still career-oriented.  **Question**: How many of the students who are career-oriented attend the economics course?  **Answer:** 208 out of 613 |

1. **Qualitative results of the eye-tracking data for research question 1 (correct Bayesian responses)**

As can be seen here in Figure 1, nodes and branches that were relevant for solving a task in a given situation (including a tree diagram as visualization) correspond very exactly and distinctly with the areas that participants looked at for the longest duration of time at. For instance, in order to answer the question “How many women who receive a negative test result have breast cancer (____ out of ____ women)?” one has to combine the second and fourth node in the lower row (i.e.,$P\left( B|\neg A \right)$ = 20 out of (8,950 + 20); see lower left of Figure 1). In fact, these two nodes turned out to be the areas that participants looked at for the longest duration by far.

*
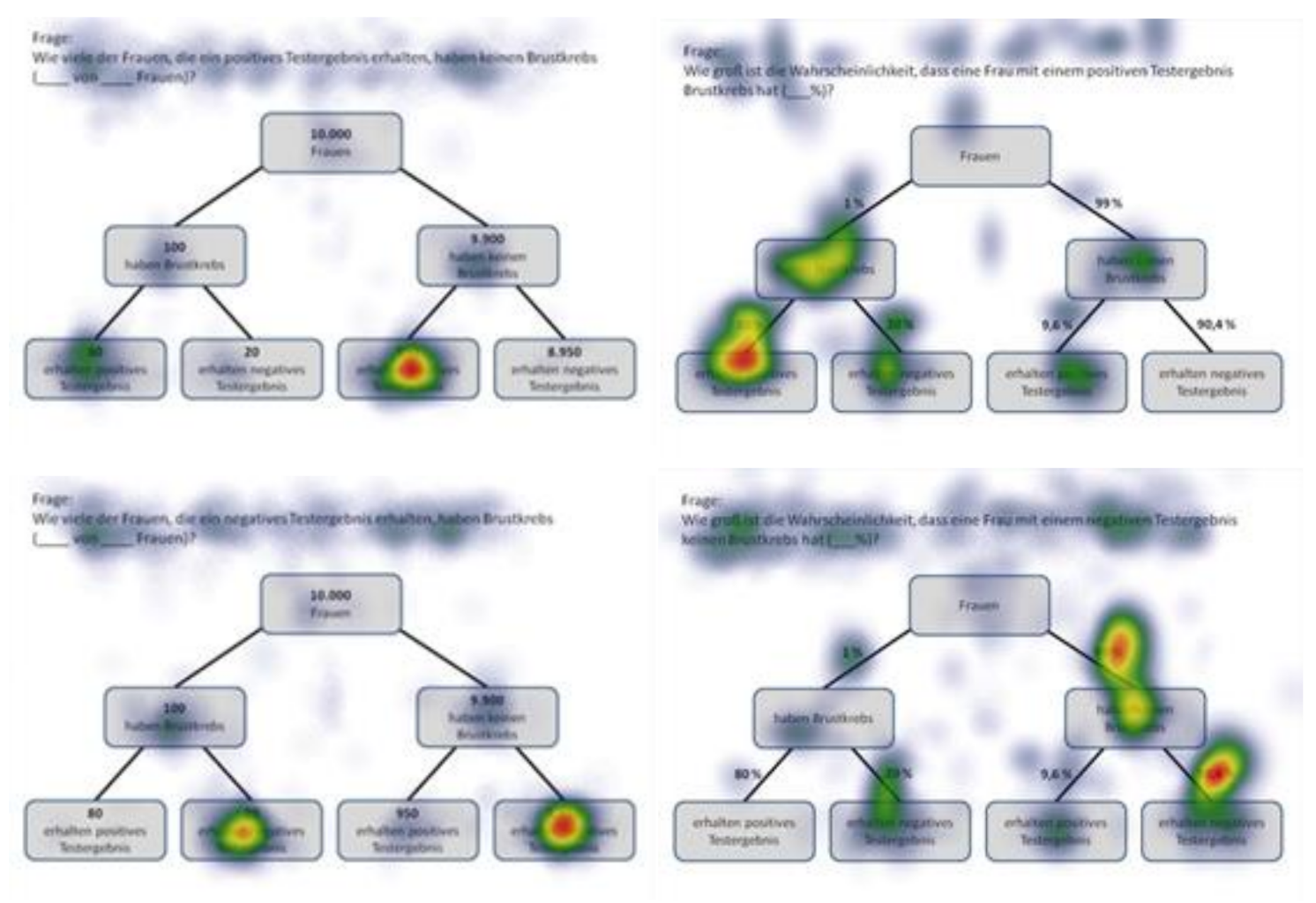
*

*Note: Colored areas indicate areas viewed in general, while red areas attracted the longest period of visual attention (on average for all 24 participants); left: absolute numbers, right: relative numbers; from upper left to lower right:*$P\left( \neg A|B \right)$*,*$P\left( A|B \right)$*,*$P\left( A|\neg B \right)$*,*$P\left( \neg A|\neg B \right)$*; original visualization in German.*

**Figure 1.** Heat maps regarding the four Bayesian inferences using 2×2 tables (if solved correctly).

Very similar to the heat map results presented in Figure 1 above, the results in Figure 2 show the four corresponding Bayesian tasks presented with 2×2 tables instead of tree diagrams. As can been seen very clearly, the results are essentially the same. For instance, to correctly answer the question “How many of the students who are not career oriented attend the economics course (____ out of ____ students)?” one has to combine the information given in the third column (i.e., $P\left( B|\neg A \right)$ = 50 out of (50 + 450); see lower left of Figure 2). Once again, task-relevant areas were those most focused on in every single case.

*
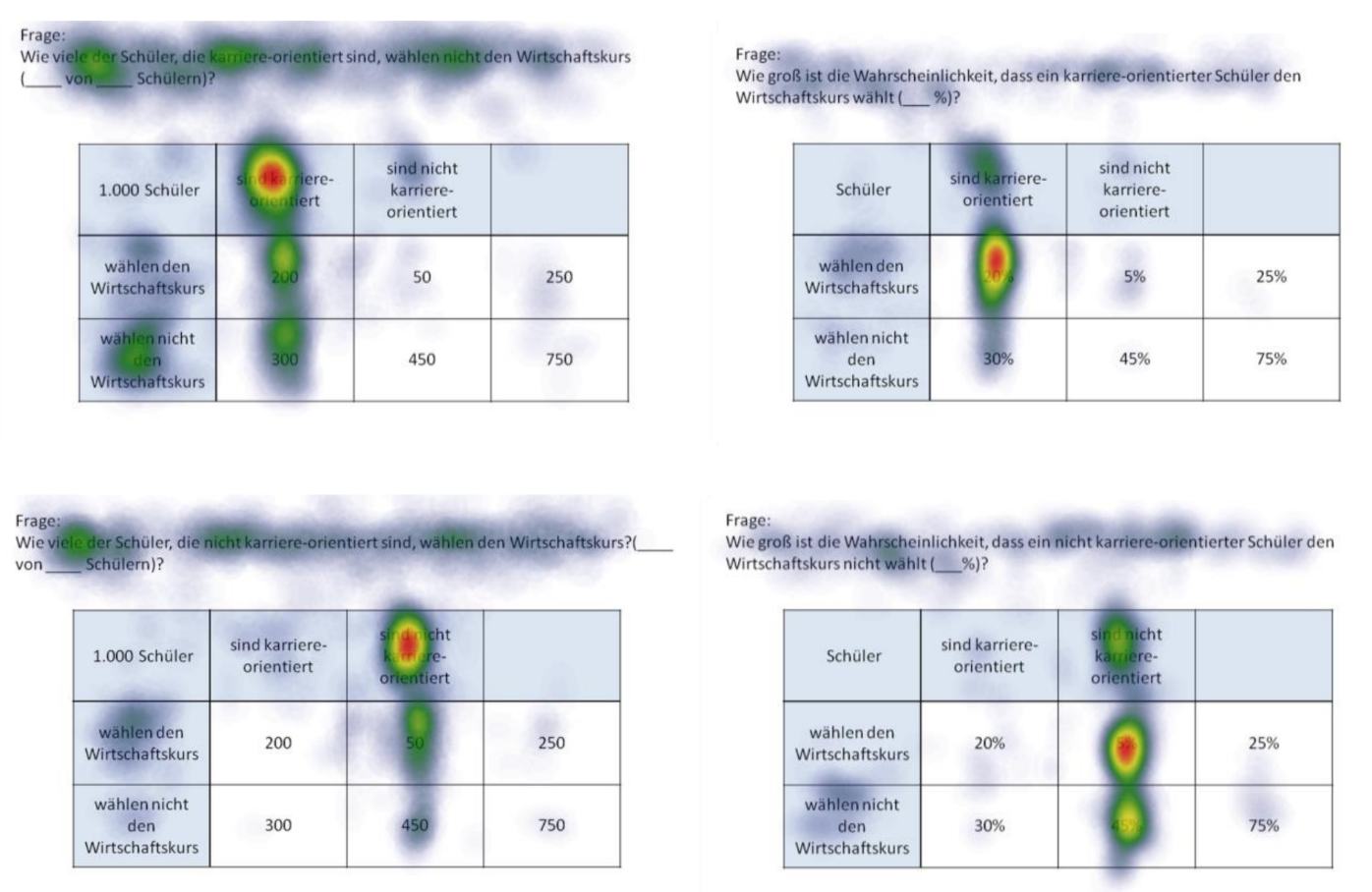
*

*Note: From upper left to lower right:* $P\left( \neg A|B \right)$*,*$P\left( A|B \right)$*,*$P\left( A|\neg B \right)$*,*$P\left( \neg A|\neg B \right)$*; original visualization in German.*

**Figure 2.** Heat maps regarding the four Bayesian inferences using 2×2 tables (if solved correctly).

1. **Qualitative results of the eye-tracking data for research question 2 (wrong responses for further Bayesian inferences)**

$P\left( A|\neg B \right),$asking for a Bayesian probability with natural frequencies, was solved correctly by *N*=11 participants (correct solution: “20 out of 8,970” = 20 out of (8,950+20) = “*E* out of (*G*+*E*)”). Participants with the correct answer again focused mainly on the relevant nodes *E* (“20”) and *G* (“8,950”) (see Figure 3). They ultimately focused more on *G* (than on *E*), which is relevant for the calculation of both the numerator and the denominator.

The heat map for wrong answer 1 (“20 out of 10,000,” *N*=5) reveals a focus on *E*, but also on *A* (“10,000”), *B* (“100”), and—only very slightly—on *G*. Obviously, participants with such scan paths not only considered a wrong subset (“10,000”), but also calculated the wrong (marginal) probability *P(*$\neg$*B)* (=joint occurrence). In contrast, participants who gave wrong answer 2 (“20 out of 100,” *N*=4) looked most intently at node *E* (as well as *B*, *C*, and *G*). Their answer corresponds to the conditional probability $P\left( \neg B|A \right)$(=Fisherian). Finally, scan paths of wrong answer 3 (“950 out of 9,900,” *N*=2) focused especially on *C* (“9,900”) and *F* (“950”). This answer erroneously corresponds to the conditional probability $P\left( B|\neg A \right)$ (=Fisherian + misreading), which means that participants giving this answer not only misunderstood the calculation algorithm but also misread the question twice. In addition, participants with wrong answers (exception: wrong answer 3) focused more on the instruction above the visualization than participants who gave the correct answers did.


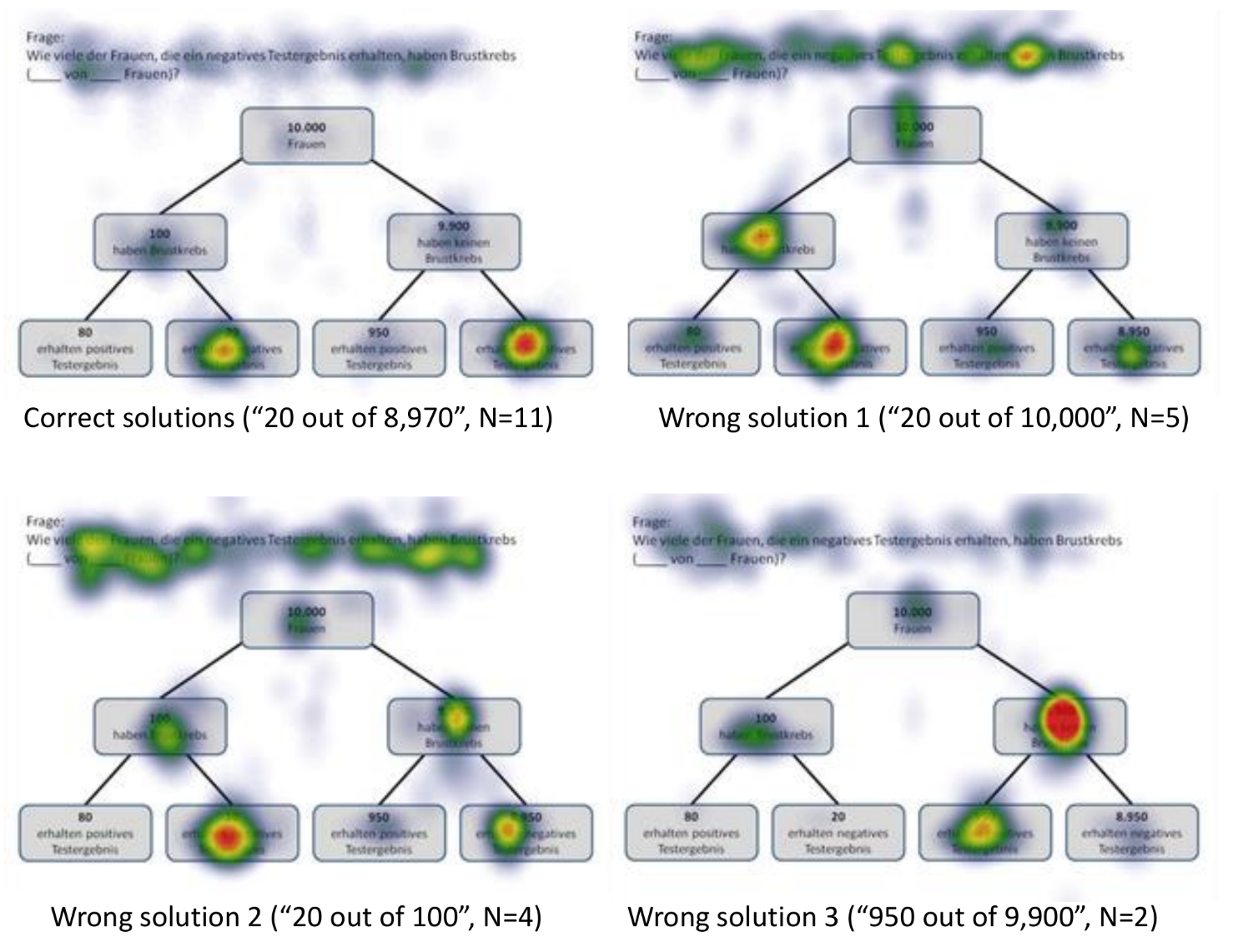


**Figure 3.** Heat maps regarding the Bayesian inference $P\left( A|\neg B \right)$ (depending on answer) with tree diagrams.

The Bayesian task $P\left( A|B \right)$ in probability format was solved correctly by only one participant (correct solution: “0.83%” ≈ 1%∙80%/(1%∙80% + 99%∙9.6% ) = “(*b*∙*d*)/(*b*∙*d*)+(*c*∙*f*)”). That participant focused mainly on the relevant branch d (“80%”) and the corresponding node *D* (“receive a positive test result”), in addition to the other relevant branches (*d*, *c*, and *f*) and nodes (*D*, *C*, and *F*), respectively. In doing so, this participant focused most on *d* (and then *b*), which is relevant for the calculation of both the numerator and the denominator.

With respect to all of the wrong answers (*N*=23, see Figure 4), the heat map results reveal a focus directed particularly at the (relevant) branches *d* and *f*. Participants who gave wrong answer 1 (“80%,” *N*=7) focused mainly on branch *d* (and then *b*, as well as on the corresponding nodes *B* and *D*). According to their answer, these participants obviously thought that they had found the correct answer as already given in branch *d* (=Fisherian)*,* which is why they more or less ignored the other (relevant) branches and nodes. In doing so, they gave an answer that erroneously represented a conditional probability. With respect to wrong answer 2 (“(about) 90%,” *N*=3), participants who chose that answer especially focused on branches *d* and *f.* They seem to have simply added up “80%” and “9.6%” (“receive a positive test result,” branches *d* and *f,* =“likelihood addition” =*”d+f”*). In addition, it is clear from the heat map that they read the question very intensively. Two other participants answered “(about) 2%” (wrong answer 3). While they also focused mainly on branches *d* and *f*, their computation that led to that result is somehow elusive.

**
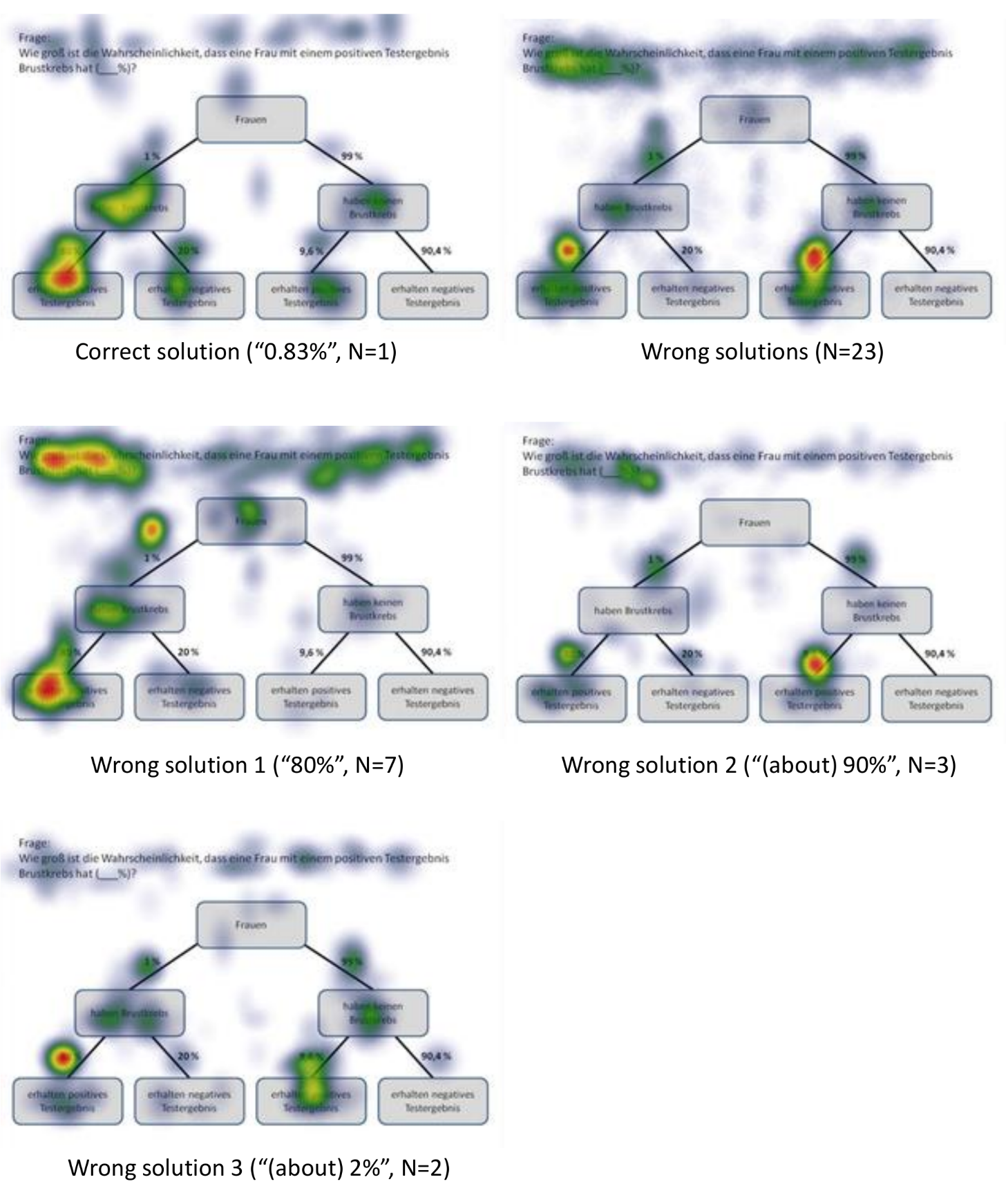
**

**Figure 4.** Heat maps regarding the Bayesian inference $P\left( A|B \right)$ (depending on answer) with tree diagrams.

$P\left( A|\neg B \right)$, asking for a Bayesian probability with natural frequencies, was solved correctly by *N*=19 participants (correct solution: “50 out of 500” = 50 out of (50+450) = “*E* out of (*E*+*G*)”). Participants with the correct answer also focused mainly on the relevant nodes *E* (“50”) and *G* (“450”) (see Figure 5). In doing so, they focused more on *G* (than on *E*), which is relevant for the calculation of both the numerator and the denominator (aside from the mere size of the number). Moreover, they also focused heavily on the marginal cell “are not career oriented,” which represents the relevant condition.

The heat map of all wrong answers (*N*=5) reveals a focus on *E* (“50”) but also on the marginal cells “are not career oriented” and “attend the economics course.” Obviously, these participants considered a wrong subset (“1,000”) since they did not focus on *G* (“450”) at all. Very similarly, scan paths from wrong answer 3 (“50 out of 1,000,” *N*=2) also show a focus especially directed at *E* in addition to the corresponding marginal cells “attend the economics course” and “are not career oriented.” Interestingly, they did not focus on *G* (“450”) at all, while they focused slightly on the total amount, “1,000 students,” which is relevant for their erroneous marginal probability answer (=joint occurrence).

**
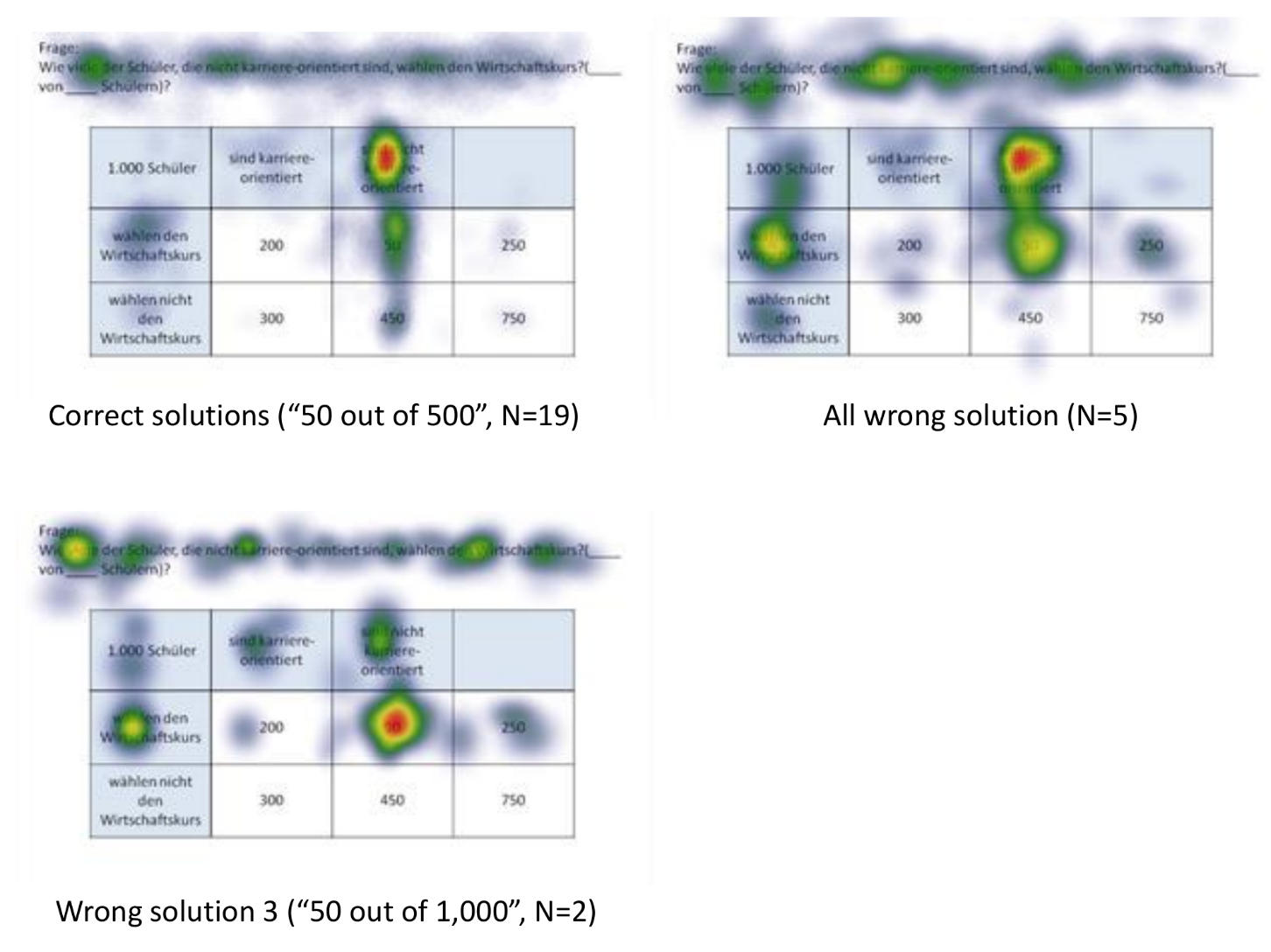
**

**Figure 5.** Heat maps regarding the Bayesian inference $P\left( A|\neg B \right)$ (depending on answer) using 2×2 tables.

Finally, the Bayesian task $P\left( A|B \right)$ was posed in probability format. The task was solved correctly by only *N*=9 participants (correct solution: “40%” = 20% / (20%+30%) = “*h* / (*h*+*j*)”). Participants with the correct answer focused mainly on the relevant cell *h* (“20%”) and the corresponding marginal cells “are career oriented” and “attend the economics course” (see Figure 6). In doing so, they focused much more on *h* (than on *j*), which is relevant for the calculation of both the numerator and the denominator.

For all wrong answers (*N*=15), the heat map reveals a stronger focus on the above-mentioned marginal cells (“attend the economics course” and “are not career oriented”). The same holds true for the most relevant mistake, wrong answer 1 (“20%,” *N*=12): Obviously, these participants thought that they had found the correct answer as already given in cell *h* (“20%”), which is why they more or less ignored the (relevant) cell *j* (“30%”). By doing so, they gave an answer that erroneously represents a conjoint probability (=joint occurrence).

**
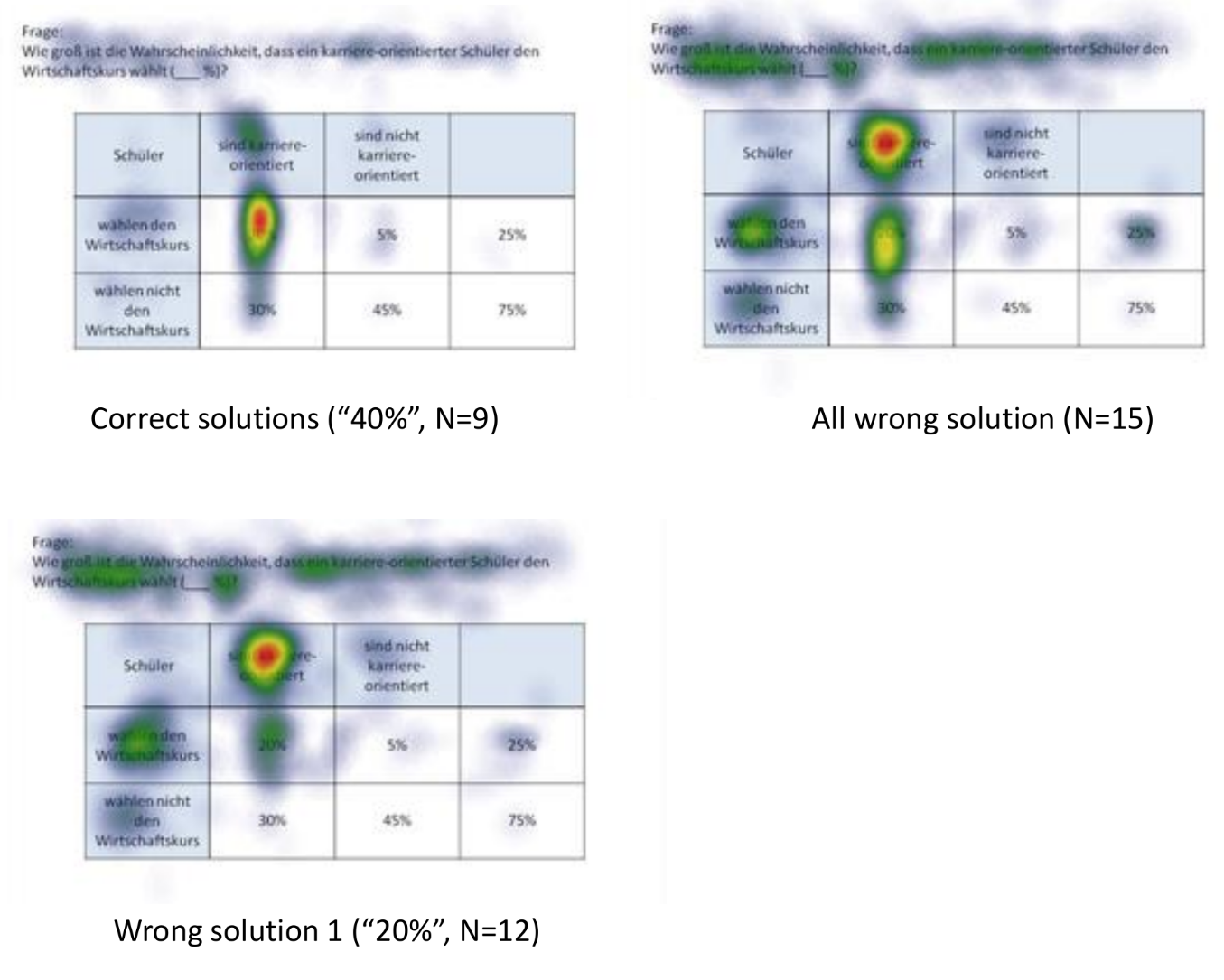
**

**Figure 6.** Heat maps regarding the Bayesian inference $P\left( A|B \right)$ (depending on answer) with 2×2 tables.
